# MoYvh1 subverts rice defense through functions of ribosomal protein MoMrt4 in *Magnaporthe oryzae*

**DOI:** 10.1371/journal.ppat.1007016

**Published:** 2018-04-23

**Authors:** Xinyu Liu, Jie Yang, Bin Qian, Yongchao Cai, Xi Zou, Haifeng Zhang, Xiaobo Zheng, Ping Wang, Zhengguang Zhang

**Affiliations:** 1 Department of Plant Pathology, College of Plant Protection, Nanjing Agricultural University, Nanjing, China; 2 Key Laboratory of Integrated Management of Crop Diseases and Pests, Ministry of Education, Nanjing, China; 3 Departments of Pediatrics, and Microbiology, Immunology, and Parasitology, Louisiana State University Health Sciences Center, New Orleans, Louisiana, United States of America; Purdue University, UNITED STATES

## Abstract

The accumulation of the reactive oxygen species (ROS) in rice is important in its interaction with the rice blast fungus *Magnaporthe oryzae* during which the pathogen scavenges ROS through the production of extracellular enzymes that promote blast. We previously characterized the MoYvh1 protein phosphatase from *M*. *oryzae* that plays a role in scavenging of ROS. To understand the underlying mechanism, we found that MoYvh1 is translocated into the nucleus following oxidative stress and that this translocation is dependent on MoSsb1 and MoSsz1 that are homologous to heat-shock protein 70 (Hsp70) proteins. In addition, we established a link between MoYvh1 and MoMrt4, a ribosome maturation factor homolog whose function also involves shuttling between the cytoplasm and the nucleus. Moreover, we found that MoYvh1 regulates the production of extracellular proteins that modulate rice-immunity. Taking together, our evidence suggests that functions of MoYvh1 in regulating ROS scavenging require its nucleocytoplasmic shuttling and the partner proteins MoSsb1 and MoSsz1, as well as MoMrt4. Our findings provide novel insights into the mechanism by which *M*. *oryzae* responds to and subverts host immunity through the regulation of ribosome biogenesis and protein biosynthesis.

## Author summary

ROS accumulation is important for the interaction between the blast fungus *M*. *oryzae* and its rice host. The protein phosphatase MoYvh1 affects the scavenging of host-derived ROS that promotes *M*. *oryzae* infection. We found that MoYvh1 is translocated to the nucleus under oxidative stress by a mechanism that is dependent on its interactions with MoSsb1 and MoSsz1. MoYvh1 triggers the release of MoMrt4 from the ribosome in the nucleus that contributes to ribosome maturation. Importantly, we have provided evidence to demonstrate that MoYvh1 is important for the synthesis of extracellular proteins that are involved in ROS scavenging. Our findings provide insight into the mechanism by which *M*. *oryzae* responds to host immunity through MoYvh1 that regulates ribosome function to evade the host defense response.

## Introduction

*Magnaporthe oryzae* is the causal agent of rice blast and also an established model organism to study plant-pathogen interactions [1,2]. In a previous study, we have characterized MoYvh1 as a homolog of the budding yeast *Saccharomyces cerevisiae* protein phosphatase Yvh1 that regulates growth, sporulation, and glycogen accumulation [3]. We found that MoYvh1 not only plays a similar important role in vegetative growth and conidia formation but also regulates virulence [4]. In addition, we found that deletion of *MoYVH1* results in an increased accumulation of the host-derived reactive oxygen species (ROS) [4]. ROS levels are known to govern the pathogen and host interaction, how MoYvh1 regulated growth and virulence is linked to its role in affecting ROS levels remains an interesting but unresolved research subject.

*S*. *cerevisiae* Yvh1 is known to also have a role in ribosome maturation and function [5]. In eukaryotic cells, mature ribosomes are composed of five different proteins that include Rpp0 and two copies of each of proteins P1 and P2 [6–8]. Rpp0 interacts directly with the 60S ribosome subunit to form the base of the stalk for binding to P1 and P2 proteins [9,10]. Also in *S*. *cerevisiae*, the ribosome assembly factor and the nucleolar protein Mrt4 are closely related to Rpp0, based on the conserved N-terminal ribosome binding domain they shared with [11,12].

Eukaryotic cells respond to environmental stresses, including elevated temperatures, via a family of well-characterized heat-shock proteins (Hsp) [13]. As ubiquitous molecular chaperones that function in a wide variety of cellular processes, Hsp70s act by reversibly binding and releasing the short hydrophobic stretches of amino acids in a nucleotide-dependent fashion [14,15]. Hsp70 heat shock proteins are known to affect ribosomal function and protein biosynthesis [16]. For example, the ribosomal L31 protein binds to chaperone Zuo1 that in turn anchors Hsp70 Ssz1 and Hsc70 proteins to regulate polypeptide translocation [17–21].

Given the multifaceted role of MoYvh1 previously established [4], further addressing of MoYvh1 functional mechanisms would promote our understanding of the rice blast mechanisms. We here showed that MoYvh1 is translocated to the nucleus under the oxidative stress condition and that MoYvh1 functions through interactions with Hsp70 protein homologs MoSsb1 and MoSsz1. In addition, we showed that MoYvh1 is required for proper translocation of the ribosomal maturation factor homolog MoMrt4, since the loss of MoYvh1 caused MoMrt4 mislocalization to the cytoplasm resulting in virulence defects.

## Results

### MoYvh1 translocates to the nucleus in response to oxidative stress

**Fig 1:**
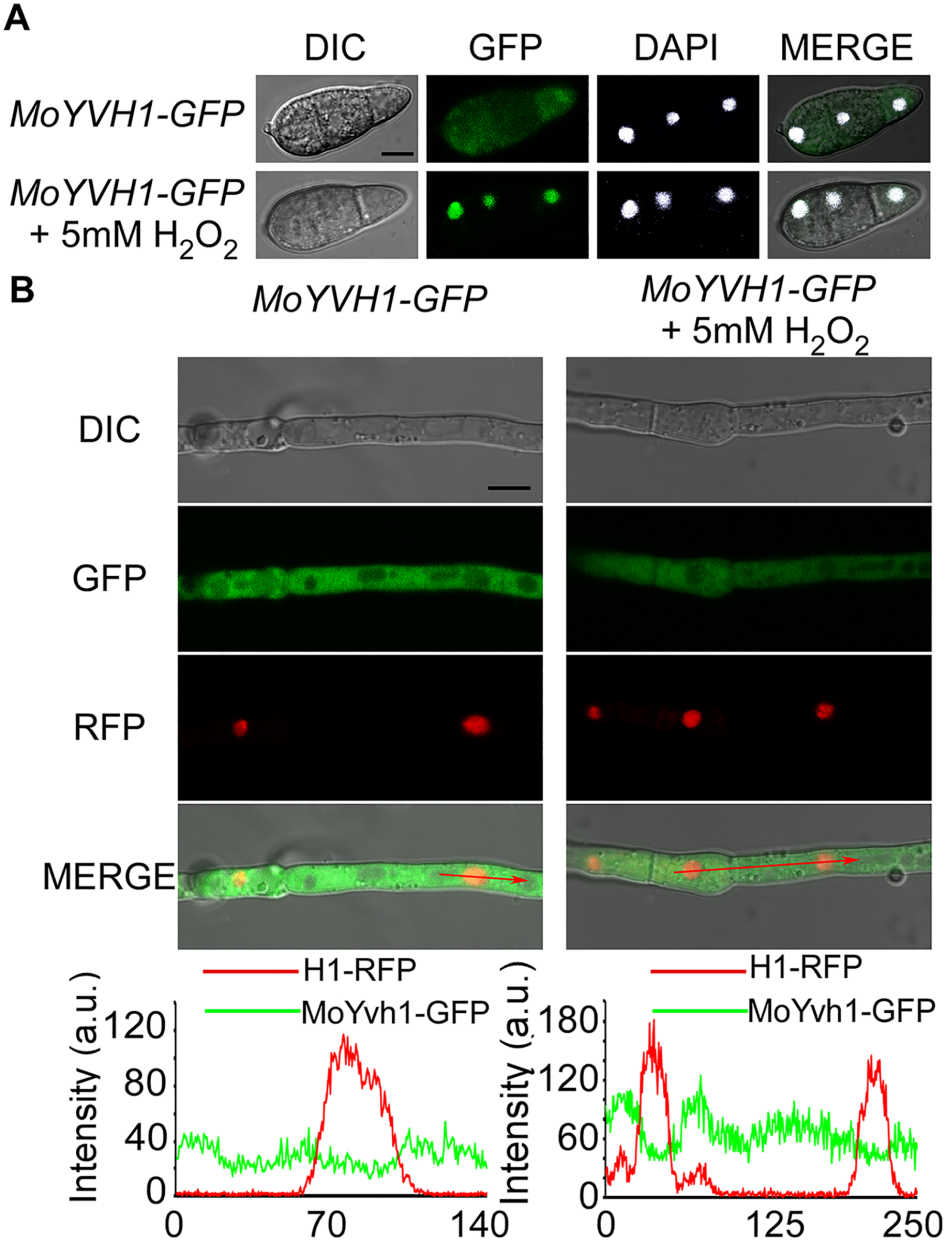
MoYvh1 translocates into the nucleus in response to the oxidative stress. (A) Fluorescence observation of conidia untreated (upper panels) and treated with 5 mM H_2_O_2_ for 2 h (lower panels). 4’,6-Diamidino-2-phenylindole (DAPI) was added to the cultures 5 min prior to the observation of the nuclei. The merged images of GFP and DAPI staining showed that MoYvh1-GFP is localized in the nucleus when treated with H_2_O_2_. Bar = 5 μm. (B) Fluorescence observation of mycelia contain MoYvh1-GFP and H1-RFP were untreated (left panels) and treated with 5 mM H_2_O_2_ for 2 h (right panels). “green line” represents MoYvh1-GFP, “red line” represents H1-RFP. Insets highlight areas analyzed by line-scan. Bar = 5 μm.

We have identified MoYvh1 as a homolog sharing amino acid sequence conservation with *S*. *cerevisiae* Yvh1 that in turn shares homology with the dual-specificity phosphatase from vaccinia virus [22]. We found that MoYvh1 has a multiple role in the growth and virulence of *M*. *oryzae* and that deletion of *MoYVH1* results in an accumulation of ROS surrounding the infection sites [4]. To address whether MoYvh1 exhibits a cytoplasmic-nuclear shuttling ability, similar to *S*. *cerevisiae* Yvh1, we constructed the strains expressing MoYvh1-GFP, in which the expression of the C-terminal GFP fusion protein is under the control of the native *MoYVH1* promoter. Notably, MoYvh1 was present in both the cytoplasm and the nucleus in conidia, which is the expected default steady-state distribution pattern. Treated with 5 mM H_2_O_2_ for 2 hours (h), an enhanced nuclear localization was observed in conidia (68.42 ± 7.31%) (Fig 1A). In the aerial hyphae, however, no changes were seen under the same stress condition (Fig 1B). Plants generate a vast array of oxidative agent in response to pathogen invasion including superoxide radical and hydroxyl radical. To further understand the changes observed in the localization pattern of MoYvh1 was specific to H_2_O_2_ or general to other oxidative stress, KO_2_ and hydroxyl radical were used to treat the Δ*Moyvh1*/*MoYVH1-GFP* strain. The results showed that both KO_2_ and hydroxyl radical induced an accumulation of MoYvh1 in the nucleus in conidia but not the aerial hyphae (S1 Fig). These data suggested that oxidative stress could promote cytoplasmic MoYvh1 nuclear localization in conidia.

### Nuclear translocation of MoYvh1 under oxidative stress requires functions of MoSsb1 and MoSsz1

**Fig 2:**
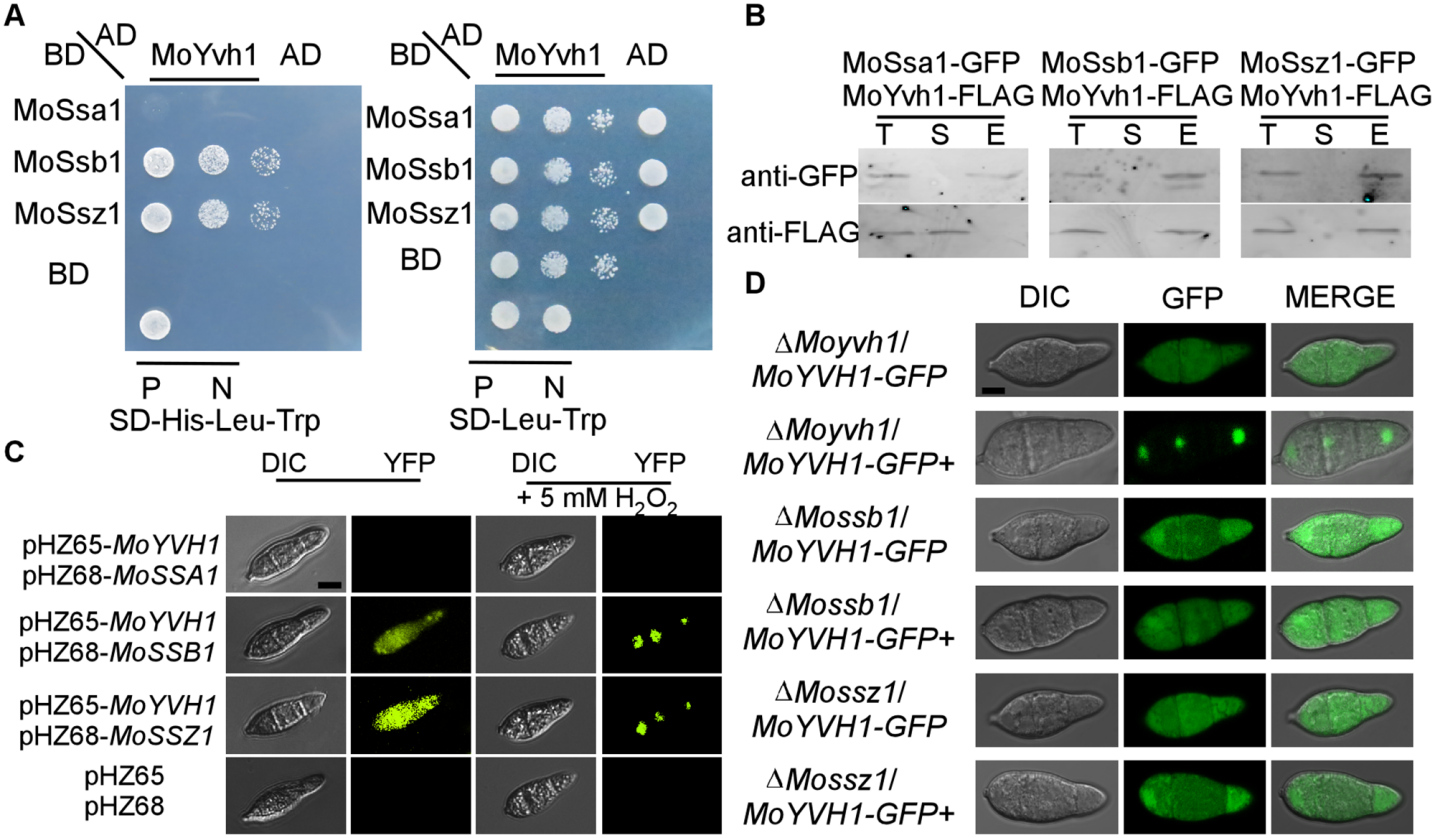
MoSsb1 and MoSsz1 are required for the translocation of MoYvh1 to the nucleus under oxidative stress. (A) Yeast two-hybrid analysis of interactions between MoYvh1 and MoSsa1, MoSsb1, and MoSsz1. MoYvh1 cDNA was inserted into the vector pGADT7, whilst MoSsa1, MoSsb1, and MoSsz1 cDNA was inserted into pGBKT7. “P” represents positive controls and “N” represents negative controls. Yeast cells grown on synthetic dextrose (SD) medium lacking leucine (Leu), tryptophan (Trp) and Histidine (His) were investigated against positive and negative controls as indicated. Plates were incubated at 30°C for 3 days before being photographed. (B) Co-IP assay. Western blot analysis of total proteins (T) extracted from conidia of various transformants, suspension proteins (S) and elution proteins (E) eluted from anti-GFP beads. The presence of MoYvh1, MoSsa1, MoSsb1 and MoSsz1 was detected with the anti-GFP and anti-FLAG antibodies, respectively. (C) Bimolecular fluorescence complementation (BiFC) assays for interactions between MoYvh1 and Hsp70 homologs. Conidia of transformants expressing MoYvh1-^N^YFP and Hsp70s-^C^YFP constructs (left panels) were treated (right panels) with 5 mM H_2_O_2_ for 2 h before interference contrast (DIC) and epifluorescence microscopy. YFP, yellow fluorescent protein. Bar = 5 μm. (D) Localization of MoYvh1-GFP was visualized in conidia of the Δ*Mossb1*, Δ*Mossz1* and Δ*Moyvh1* mutants. “+” represents the samples treated with 5 mM H_2_O_2_ for 2 h. Bar = 5 μm.

To understand MoYvh1 functions associated with its nuclear translocation, we identified MoSsb1 and MoSsz1 that are heat-shock 70 (Hsp70) protein homologs following screening a yeast two-hybrid cDNA library constructed with RNA pooled from various stages including conidia and infections (0, 2, 4, 8, 12 and 24 h) (Fig 2A). We then validated these interactions by co-introducing the *MoYVH1*-FLAG and *MoHSP70s*-GFP fusion constructs into the protoplasts of the wild type strain Guy11. Total proteins were extracted from conidia of the putative transformants, and MoYvh1, MoSsa1, MoSsb1, and MoSsz1 were detected using the anti-FLAG and anti-GFP antibodies. In proteins eluted from MoSsb1 and MoSsz1 anti-GFP beads, MoYvh1 was detected (Fig 2B). The interactions were further confirmed by the bimolecular fluorescence complementation (BiFC) assay. The *MoYVH1*-^C^YFP and *MoSSB1*-^N^YFP, *MoSSZ1*-^N^YFP fusion constructs were introduced into the protoplasts of Guy11, with the empty vectors used as negative controls. The recombined YFP fluorescence signal was detected in the cytoplasm containing corresponding protein pairs (Fig 2C and S2 Fig). Interestingly, the interactions were also observed under the oxidative stress, with YFP fluorescence being transferred to the nucleus following treatment with 5 mM H_2_O_2_ (Fig 2C). Previously, we demonstrated that the MoYvh1 C-terminal zinc-binding domain is required for growth and virulence of the fungus. Our evidence here showed that the same C-terminal is also responsible for binding with MoSsb1 and MoSsz1 (S3 Fig).

An interaction between MoYvh1 and MoSsa1 could not be established, suggesting that MoYvh1 interactions with MoSsb1 and MoSsz1 are specific (Fig 2A, 2B and 2C). We also generated *MoSSB1* and *MoSSZ1* deletion mutants and assessed their effects on MoYvh1 distribution. The *MoYVH1-GFP* fusion construct was introduced into Δ*Mossb1*, Δ*Mossz1*, and Δ*Moyvh1* mutants. In the resulting transformants, GFP signal was observed in both the cytosol and the nucleus. However, the GFP signal was predominantly observed in the cytoplasm of Δ*Mossb1* and Δ*Mossz1* upon oxidative stress, in contrast to complemented strains (72.16 ± 5.77%) where GFP was predominantly seen in the nuclei (Fig 2D). To further evaluate the nuclear translocation of MoYvh1 in these strains, we separated nuclear proteins from cytoplasmic ones and performed Western blotting analysis. MoYvh1-GFP was significantly enriched in the nucleus in the complement strain when treated with 5 mM H_2_O_2_. However, MoYvh1-GFP was uniformly distributed in the conidia of the Δ*Mossb1* and Δ*Mossz1* mutants (S4 Fig). These results suggested that MoSsb1 and MoSsz1 could facilitate nuclear translocation of MoYvh1 under oxidative stress through direct interactions.

### MoMrt4^G69D^ and MoMrt4^G69E^ suppress the defects of Δ*Moyvh1* mutants in growth and virulence

**Fig 3:**
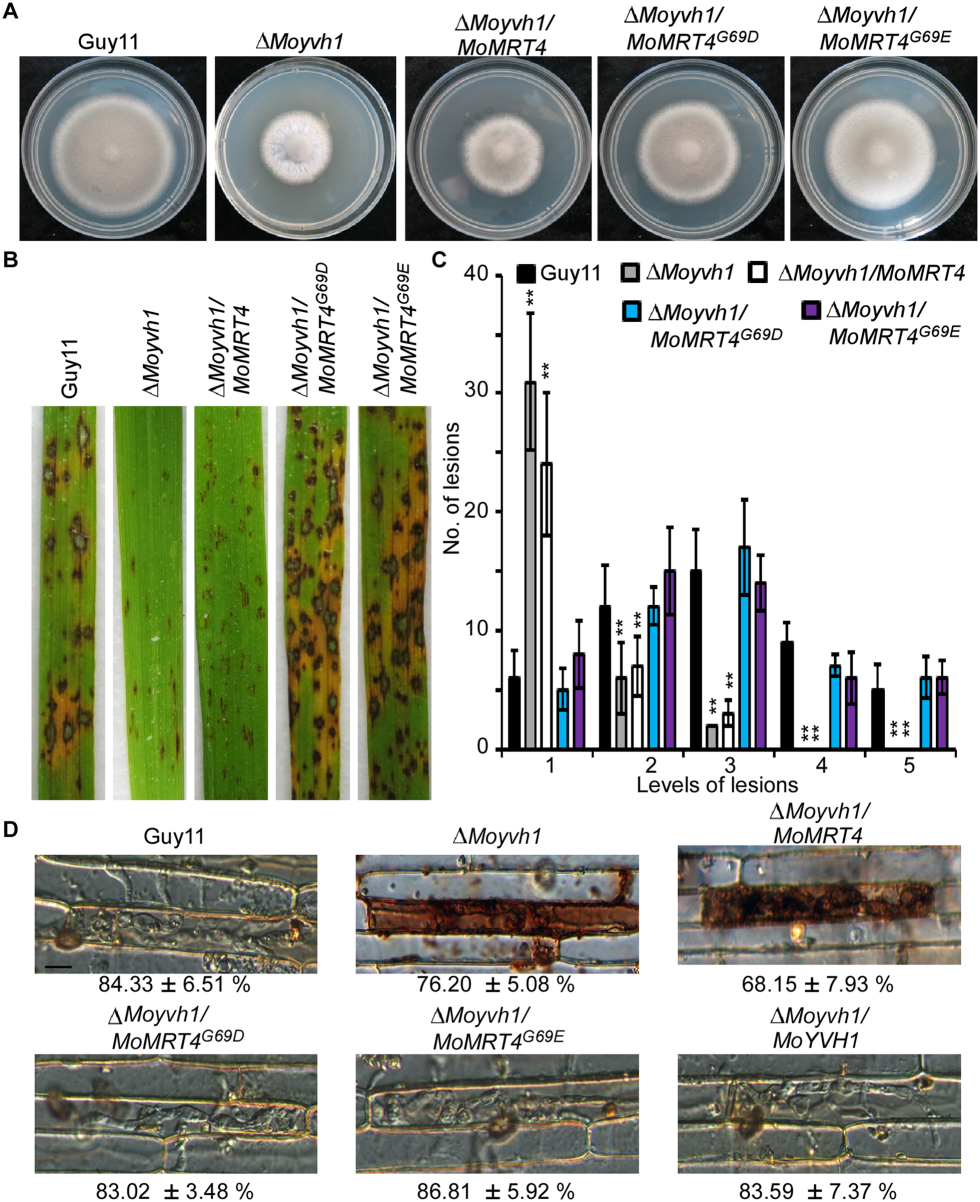
MoMrt4^G69D^ and MoMrt4^G69E^ bypass the requirement of MoYvh1. (A) Comparison of the Δ*Moyvh1* and point mutation strains in colony morphology. Guy11, the Δ*Moyvh1* mutant, *MoMRT4* and mutated alleles complementation strains were cultured at 28°C in darkness for 7 days before being photographed. (B) Spraying assay. Conidial suspensions of each strain were sprayed on rice seedlings. Diseased leaves were photographed 7 days after inoculation. (C) Quantification of lesion type. Quantification of lesion type (0, no lesion; 1, pinhead-sized brown specks; 2, 1.5-mm brown spots; 3, 2–3-mm grey spots with brown margins; 4, many elliptical grey spots longer than 3 mm; 5, coalesced lesions) were measured at 7 days post-inoculation (dpi), counted within an area of 4 cm^2^ and experiments were repeated three times with similar results. Asterisk indicates significant differences at p = 0.01. (D) DAB staining of the excised leaf sheath of rice infected by Guy11, the Δ*Moyvh1* mutant, the point mutation strains and complementation strain 36 h after inoculation. Bar = 5 μm.

To further understand MoYvh1 nuclear translocation upon stress and associated functions, we searched for additional proteins that interact with MoYvh1 and identified MoMrt4 (MGG_08908) that shares sequence homolog with *S*. *cerevisiae* nucleolar protein Mrt4. The yeast Mrt4 contains a Gly residue at position 68 whose substitution with Asp or Glu could suppress the growth defect of the Δ*yvh1* strain [5,23,24]. To investigate whether MoYvh1 shares functional conservation with *S*. *cerevisiae* Mrt4, we constructed strains expressing *MoMRT4*^*G69D*^*-GFP* and *MoMRT4*^*G69E*^*-GFP*, respectively. We found that MoMrt4^G69D^ and MoMrt4^G69E^, but not MoMrt4, were able to rescue the defect on growth and virulence of the Δ*Moyvh1* strain (Fig 3A, 3B and 3C and S5A Fig). Because MoYvh1 functions upstream of MoPdeH to regulate the cAMP levels and pathogenicity [4], we also found that MoMrt4^G69D^ and MoMrt4^G69E^ suppress the defects in cAMP levels of the Δ*Moyvh1* mutant (S5B Fig).

As MoYvh1 plays a role in scavenging host-derived ROS, we examined ROS levels by staining host cells with 3, 3’-diaminobenzidine (DAB) at 36 h after inoculation. The primary infected rice cells with infectious hyphae of the Δ*Moyvh1* and Δ*Moyvh1/MoMRT4* strains were stained intensely by DAB, with reddish-brown precipitate around the infected cells, while the Δ*Moyvh1/MoMRT4*^*G69D*^ and Δ*Moyvh1/MoMRT4*^*G69E*^ strains exhibited weak staining, a phenotype similar to Guy11 (Fig 3D).

### MoYvh1 competes with MoMrt4 onto the ribosome for its function

**Fig 4:**
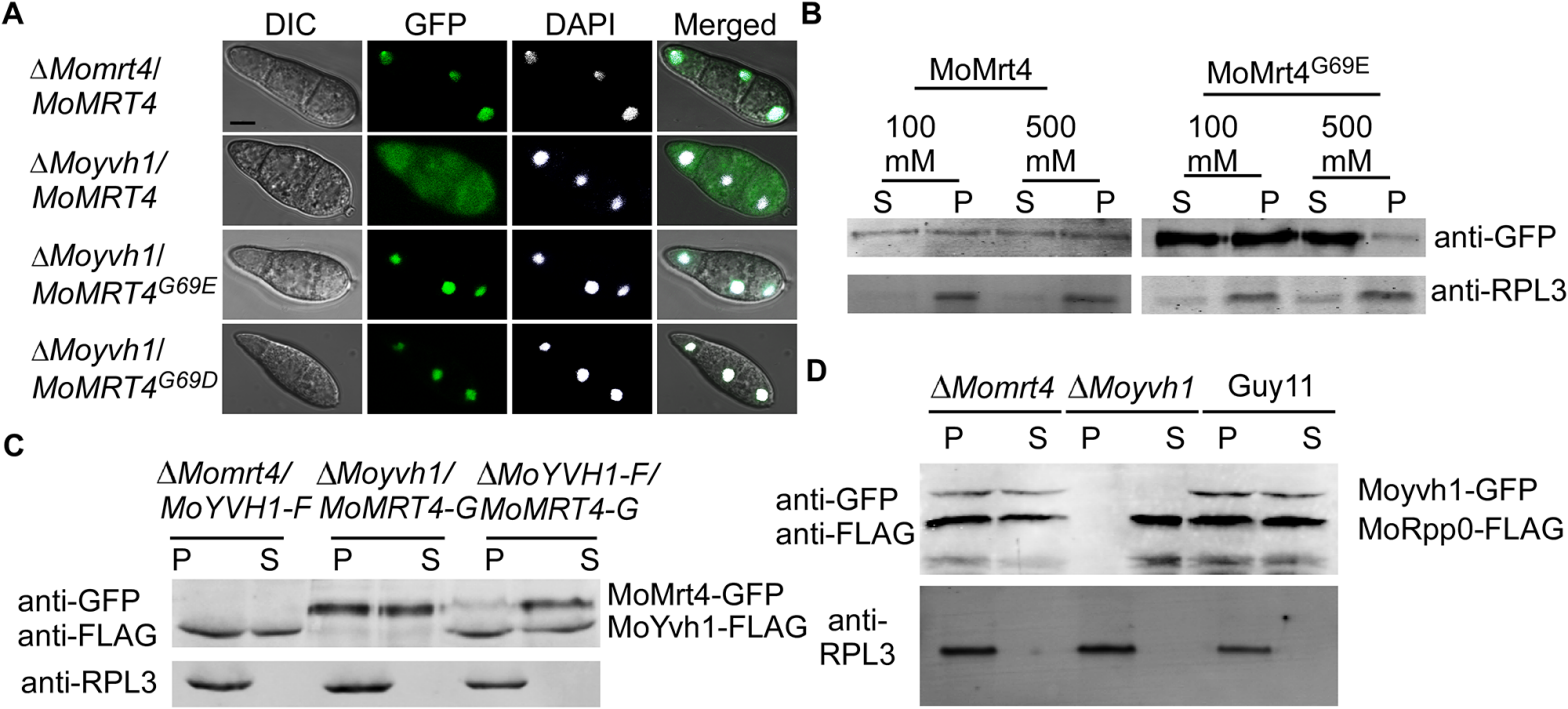
Dissociation of MoMrt4 from the pre-ribosome in the nucleolus facilitated by MoYvh1 is required for the ribosome maturity. (A) The localization of Mrt4-GFP, MoMrt4^G69D^-GFP and MoMrt4^G69E^-GFP was observed in conidia of the Δ*Momrt4* and Δ*Moyvh1* mutants. DAPI was used to stain nuclei. The merged image of GFP and DAPI staining showed that Δ*Moyvh1*/*MoMRT4–GFP*, Δ*Moyvh1*/*MoMRT4*^*G69D*^*-GFP* and Δ*Moyvh1*/*MoMRT4*^*G69E*^*-GFP* strains were localized in the nucleus. Bar = 5 μm. (B) Ribosomal proteins were prepared from Δ*Moyvh1*/*MoMRT4–GFP* and Δ*Moyvh1/MoMRT4*^*G69E*^*-GFP* strains grown at as indicated salt concentrations. Free and ribosome-bound proteins were separated by sedimentation through sucrose cushions. Equal amounts of supernatant (S) and pellet (P) were separated by SDS-PAGE, and the presence of MoMrt4 and Rpl3 (a ribosome marker) was detected by Western blotting analysis using anti-GFP or anti-RPL3 antibodies. (C) Ribosome proteins were extracted from the strains as indicated. Free and ribosome-bound proteins were separated by sedimentation through sucrose cushions. Both the anti-GFP and anti-FLAG antibodies were added to detect the presence of MoMrt4 and MoYvh1 in supernatants (S) and pellets (P) following SDS-PAGE. RPL3 was used as a marker for ribosome. (D) Ribosome proteins of the indicated strains were extracted. Equal amounts of supernatant (S) and pellet (P) were separated by SDS-PAGE, and MoRpp0 and MoYvh1 were detected by Western blotting using anti-GFP and anti-FLAG antibodies.

MoMrt4 is normally accumulated in the nucleus of the wild-type strain (Fig 4A). To study how MoMrt4^G69D^ and MoMrt4^G69E^ suppress the defects of the Δ*Moyvh1* mutant, we assessed the effect of MoYvh1 on the subcellular localization of MoMrt4. As expected, MoMrt4^G69D^ and MoMrt4^G69E^ were predominantly nuclear localized, while MoMrt4 was mostly cytoplasmic, in the Δ*Moyvh1* mutant (Fig 4A). As MoMrt4^G69D^ and MoMrt4^G69E^ mutation showed similar roles in the Δ*Moyvh1* mutant, we used the Δ*Moyvh1*/*MoMrt4*^*G69E*^ strain to determine whether its affinity for the ribosome was compromised. We found that binding of MoMrt4^G69E^ to the ribosome was more sensitive to 100 and 500 mM NaCl than MoMrt4 that was largely unaffected. 500 mM NaCl caused the majority of MoMrt4^G69E^ to be dissociated from the ribosome (Fig 4B). Therefore, MoMrt4^G69E^ showed weaker affinity for ribosomes than MoMrt4, implying easier separation from the ribosome. We further speculated that the affinity for ribosomes between MoYvh1 and MoMrt4 is important for the normal function of *M*. *oryzae*. To test this hypothesis, we assessed whether MoMrt4 competes with MoYvh1 in ribosome binding. Western blotting analysis showed that MoYvh1 bound to the ribosome in both the wild-type and the Δ*Momrt4* mutant. However, the MoMrt4 recruitment to ribosomes in the presence of MoYvh1 was significantly reduced in the wild-type strain (Fig 4C), suggesting that MoYvh1 and MoMrt4 indeed compete for binding to the ribosome.

The Rpp0 protein is one of the five conserved components of mature ribosomes [7,10]. We have cloned the MoRpp0 homolog and generated the Δ*Moyvh1*/MoRpp0-FLAG-MoYvh1-GFP, Δ*Momrt4*/MoRpp0-FLAG-MoYvh1-GFP, and Guy11/MoRpp0-FLAG-MoYvh1-GFP strains to investigate whether deletion of MoYvh1 or MoMrt4 causes any defects in ribosome maturity. Ribosome proteins were extracted. In the Δ*Momrt4* mutant, MoRpp0 was bound to the ribosome similar to that in the wild-type strain. However, MoRpp0 remained in the suspension in the Δ*Moyvh1* mutant (Fig 4D), suggesting that MoYvh1 has a role in ribosome maturity.

### MoMrt4 is important for growth, development, and virulence

**Fig 5:**
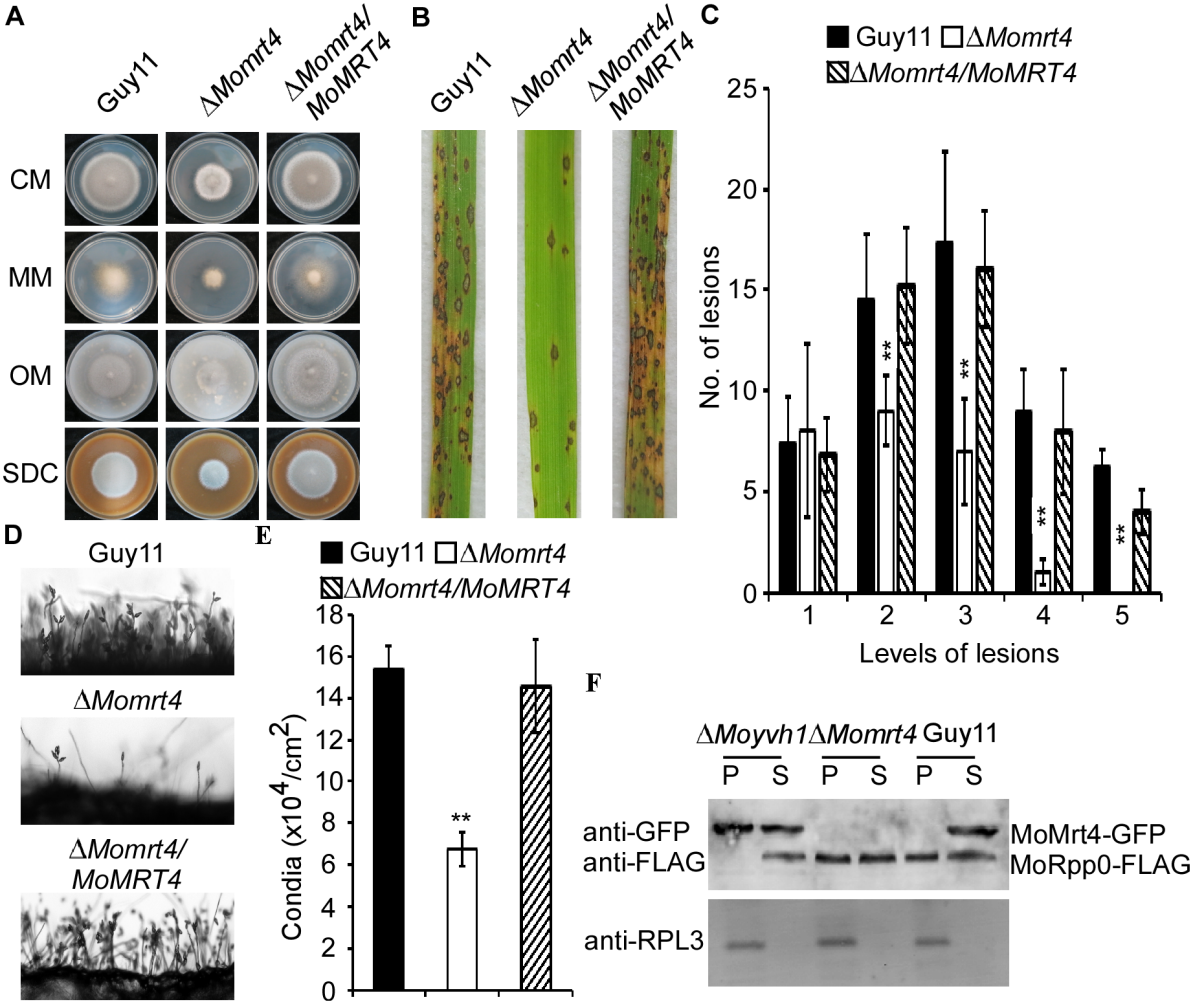
MoMrt4 is important for vegetative growth, conidiation, and full virulence. (A) Comparison of the wild type, the Δ*Momrt4* and the complement strains in vegetative growth on various medium. (B) The pathogenicity assay on rice leaves. Conidial suspensions of strains were sprayed onto two-week old rice seedlings (CO-39). Diseased leaves were photographed after 7 days of inoculation. (C) Quantification of lesion type. Lesions were photographed and measured at 7 days post-inoculation (dpi), counted within an area of 4 cm^2^ and experiments were repeated three times with similar results. Asterisk indicates significant differences at p = 0.01. (D) Conidia development of the wild type, Δ*Momrt4* and the complement strains on SDC medium for 7 days were examined by light microscopy. (E) Statistical analysis of conidia production. Conidia produced by the wild-type, the mutant and complemented strains on SDC medium for 10 days were collected, counted and analyzed. ±SD is calculated from three repeated experiments and asterisks indicate statistically significant differences (Duncan’s new multiple range test, p < 0.01). (F) Ribosome proteins of indicated strains were extracted. Equal amounts of supernatant (S) and pellet (P) were separated by SDS-PAGE, and MoRpp0 and MoMrt4 were detected by Western blotting analysis using the anti-GFP and anti-FLAG antibodies, respectively.

Since MoMrt4 is important for MoYvh1 function, we characterized its function in growth and pathogenesis. The Δ*Momrt4* mutant displayed significantly attenuated growth on CM, minimal medium (MM), straw decoction and corn agar (SDC), and oatmeal medium (OM) plates (Fig 5A and S5C Fig). Conidia formation was drastically reduced in the Δ*Momrt4* mutants by ~70% when compared with the wild-type strain (Fig 5D and 5E). To determine whether MoMrt4 plays a role in pathogenicity, susceptible CO-39 rice seedlings were respectively sprayed with the conidia of the wild-type, Δ*Momrt4* mutant, and complemented strains. The production of fewer, small lesions by the Δ*Momrt4* mutant at 7-day post-inoculation (dpi) (Fig 5B and 5C) indicated that MoMrt4 is required for full virulence.

Our previous study showed that deletion of *MoYVH1* results in an increase in the accumulation of ROS but reduced virulence [4]. To test that the reduced virulence was due to a lack of ROS scavenging, we examined host-derived ROS levels by DAB staining. At 30 h after inoculation, no staining was observed in the primary rice cells infected by the Δ*Momrt4* mutant (S6 Fig). We also evaluated binding of MoMrt4 to ribosomes in these strains and found that MoRpp0 remained in the suspension of the Δ*Moyvh1* mutant. However, MoRpp0 bound to the ribosome in the Δ*Momrt4* mutant which was similar to that in Guy11, indicating that deletion of *MoMRT4* was not involved in the ribosome maturity, in contrast to MoYvh1 (Fig 5F). These results revealed that MoMrt4 is required for vegetative growth, conidiation, and full virulence, but these functions are independent of ribosome maturity.

### MoYvh1 is nuclear translocated in response to ROS

**Fig 6:**
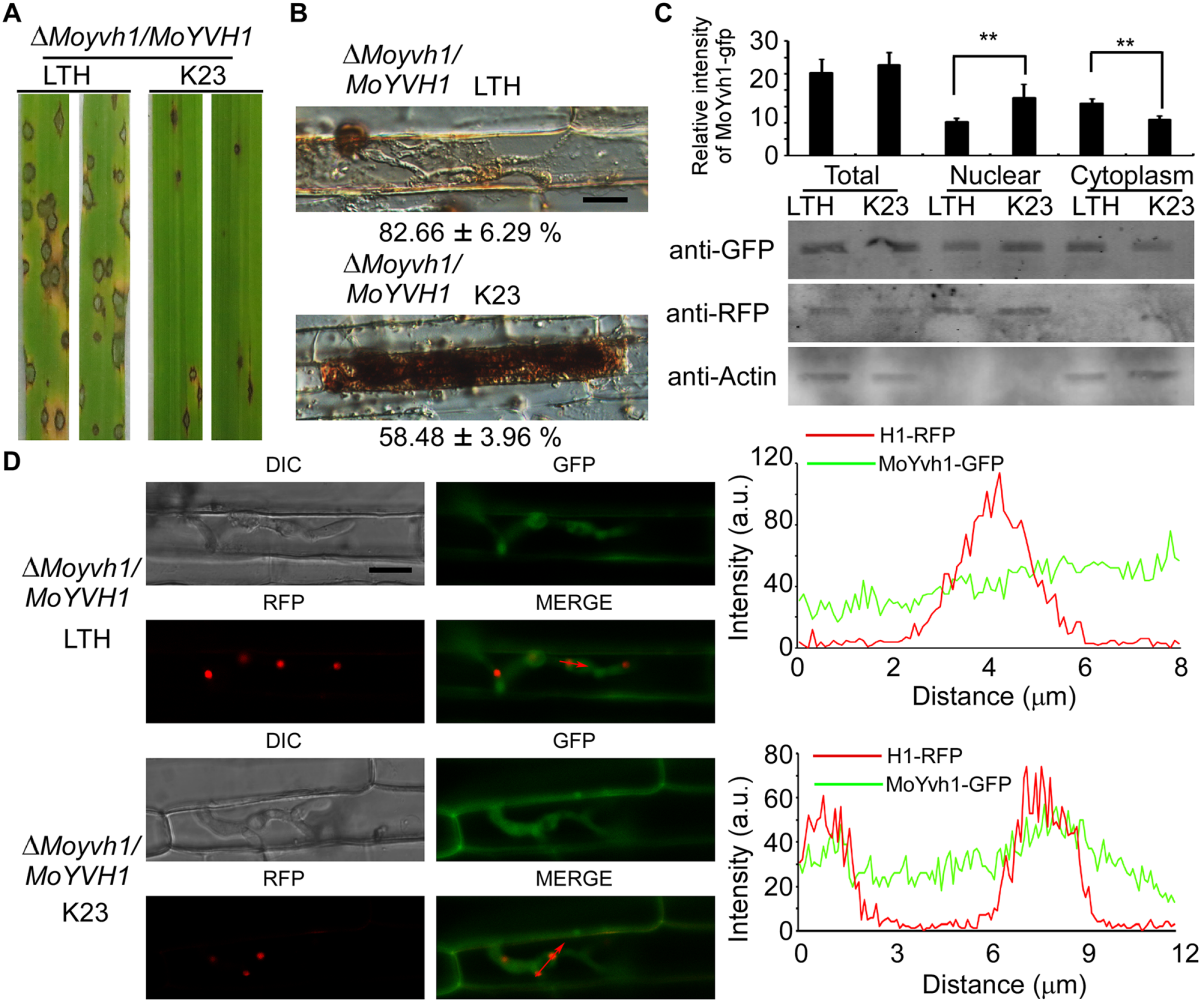
Host derived ROS induces MoYvh1 nuclear accumulation during infection. (A) Whole-plant assays with cultivars LTH and K23 inoculated with the Δ*Moyvh1/MoYVH1* strain. The strain formed rare, small lesions on K23 which were different from the lesions on LTH. Plants were photographed at 7 d after inoculation. (B) DAB staining of the excised leaf sheath of cultivars LTH and K23 infected by the Δ*Moyvh1/MoYVH1* strain 30 h after inoculation. Bar = 5 μm. (C) Rice leaves were incubated with Δ*Moyvh1/MoYVH1-GFP-H1-RFP* strain for 30 h. Equal weight of rice leaves (LTH and K23) were divided into three parts for extraction of total, nuclear and cytoplasm proteins. Equal amounts of total, nuclear and cytoplasm proteins were separated by SDS-PAGE, and the presence of MoYvh1 was detected by Western blotting using the anti-GFP antibody. The intensity of Western blotting bands was quantified with the ODYSSEY infrared imaging system (application software Version 2.1). The intensity of MoYvh1 was compared between the cv. LTH and cv. K23 among total proteins, nuclear proteins, and cytoplasmic proteins. H1 (a nucleus marker) and actin (a cytoplasm marker) were detected by Western blotting analysis using the anti-RFP or anti-Actin antibodies. Bars denote standard errors from three independent experiments. Asterisk indicates significant differences (Duncan’s new multiple range test p < 0.01) (D) Localization of MoYvh1 during infection. Infection hyphae contain MoYvh1 and H1-RFP were observed by confocal fluorescence microscopy in the sheath of cultivars of LTH and K23 at 30 hpi. “green line” represents MoYvh1-GFP, “red line” represents H1-RFP. Insets highlight areas analyzed by line-scan. Bars = 5μm.

As the nuclear localization of MoYvh1 is enhanced in conidia upon oxidative stress, we hypothesized that MoYvh1 is also translocated to the nucleus during host-imposed stress during infection. To test this, we screened rice cultivars resistant to Guy11 and the Δ*Moyvh1/MoYVH1* strains. We found that the wild type strains caused only the restricted lesions on the rice cultivar K23 that contains the resistant gene *Pi12* [25] (Fig 6A). As the Δ*Moyvh1/MoYVH1* strains showed restricted lesions on the K23, DAB was further used to evaluated the host-derived ROS accumulated around the infection sites in K23. Cells with Δ*Moyvh1/MoYVH1-GFP* infectious hyphae on rice cultivar K23 were stained by DAB, with the reddish-brown precipitate around the infected cells, indicating that the Δ*Moyvh1/MoYVH1-GFP* strain fails to scavenge H_2_O_2_ on K23 (Fig 6B). To assess nuclear translocations of MoYvh1, we extracted nuclear proteins and performed Western blotting analysis. In K23, MoYvh1-GFP was significantly enriched in the nucleus in comparison with LTH (Fig 6C). As it is difficult to stain the nucleus by DAPI during infection, we used the histone H1 fused to red fluorescent protein (RFP) to mark the nucleus of infectious hyphae. We found an enrichment of MoYvh1 in the nucleus when co-localization with H1-RFP in the infection hyphae of K23, in comparison with LTH cultivar (Fig 6D). However, MoYvh1 was not translocated into the nucleus in the Δ*Mossb1* and Δ*Mossz1* mutants (S7 Fig). These results indicated that MoYvh1 is indeed nuclear enhanced during infection.

### MoYvh1 plays a role in the biosynthesis of extracellular proteins that suppress host defense responses

**Fig 7:**
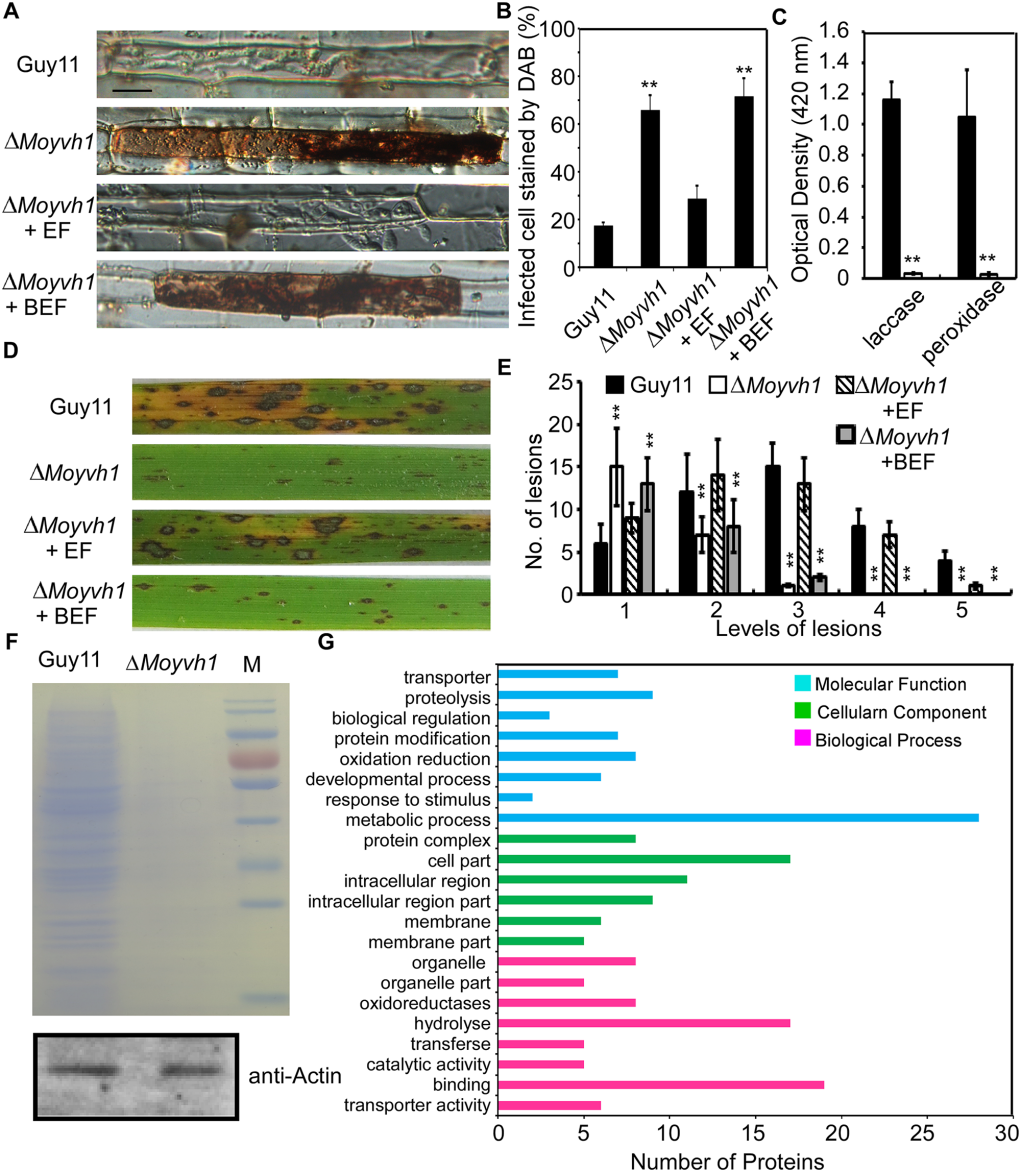
MoYvh1 nuclear localization promotes extracellular proteins to evade host innate immunity. (A) Extracellular fluids from Guy11 appressoria suppress the defects in scavenging host-derived ROS of the Δ*Moyvh1* mutant, whereas boiled EF did not exhibit a similar defense response. “EF” represents the extracellular fluid. “BEF” represents the boiled extracellular fluid. Data represents observations from three independent experiments. Bar = 5 μm. (B) The infected cell stained by DAB. Three independent biological experiments were performed, with three replicates each time and yielded similar results in each independent biological experiment. Error bars represent standard deviation, and asterisks represent significant difference between the different strains (p < 0.01). (C) Laccase activity measured by ABTS oxidizing test without H_2_O_2_ and peroxidase activity measured by ABTS oxidizing test with H_2_O_2_. Bars denote standard errors from three independent experiments. Asterisk indicates significant differences (Duncan’s new multiple range test p < 0.01) (D) The conidial suspensions of each treatment were sprayed on the rice leaves. “EF” represents the extracellular fluid. “BEF” represents the boiled extracellular fluid. (E) Quantification of lesion type. Lesions were photographed and measured at 7 days post-inoculation (dpi), counted within an area of 4 cm^2^ and experiments were repeated three times with similar results. Asterisk indicates significant differences at p = 0.01. (F) 1D gel analysis of Guy11 and the Δ*Moyvh1* mutant extracellular fluid proteins: Equal numbers of Guy11 and Δ*Moyvh1* conidia were harvested and equalized by using the actin antibody. The same number of conidia was used to extract the extracellular fluid. The extracellular fluid was concentrated to 100 μl. A 50 μl aliquot of each sample was then fractionated by 1D SDS-PAGE and proteins were stained by Coomassie brilliant blue. (G) GO functional analysis of extracellular fluid proteins.

At the early stages of infection, *M*. *oryzae* secretes numerous effector proteins to suppress plant defense responses and modulate host cellular processes that promote infections [26]. Since MoYvh1 has a role in ribosome maturity (Fig 5D) and the ribosome is important for the synthesis of proteins, we tested whether the production of extracellular proteins was compromised in the Δ*Moyvh1* mutant. The extracellular fluid (EF) was prepared as described by Patkar and colleagues [27]. Conidia from Guy11 and the Δ*Moyvh1* strains were inoculated on a hydrophobic glass sheet and EF was harvested following 24 h incubation. We first detected the localization of MoYvh1 under this condition and the results showed that MoYvh1 was present in both nucleus and cytoplasm, indicating MoYvh1 functions in the nucleus during the appressorium formation (S8 Fig). EF extracts from wild type were subsequently added to the rice leaf sheaths following infection by the Δ*Moyvh1* mutant. We found that native EF, but not that denatured by boiling, rescued the defects in host cell invasion and ROS scavenging at the infected sites (Fig 7A and 7B). Our previous study showed that deletion of *MoYVH1* resulted in reduced peroxidase and laccase activities [4], so we further assayed the peroxidase and laccase activities in both EF harvested from the wild type and Δ*Moyvh1* mutant strains. The enzyme activity assay was performed as described by Chi and associates [28] by using the EF from both Guy11 and Δ*Moyvh1* mutant. We observed very low levels of laccase and peroxidase activities in the Δ*Moyvh1* mutant when compared with Guy11 (Fig 7C). We further performed the spray and drop assays on rice leaves. Conidia of the Δ*Moyvh1* mutant were collected with 5 ml of the EF or boiled EF of Guy11. The conidial suspensions of each treatment were sprayed onto rice leaves. After inoculation for 7 days, the results showed that the EF of Guy11 could suppress the defects of the Δ*Moyvh1* mutant in pathogenicity (Fig 7D and 7E). The conidial suspensions of each treatment were also drop inoculated onto detached rice leaves and the results revealed that the EF of the wild type strain partially rescues the defect in pathogenicity on the detached rice leaves (S9 Fig). These results indicated that the EF of Guy11 contains candidate proteins that are important for infection.

To identify candidate proteins in EF regulated by MoYvh1, we compared the EF production through SDS-PAGE analysis and found that the amount of EF proteins in Δ*Moyvh1* EF was significantly less than that of wild type Guy11 (Fig 7F). In addition, mass spectrometry (MS) analysis revealed the presence of over 70 proteins with signal peptides in EF of the wild type strain but not of the Δ*Moyvh1* mutant. To address whether the absence of these proteins is caused by the defect in ribosomal biogenesis, we randomly chosen 30 of them to evaluate the transcriptional difference between Guy11 and the Δ*Moyvh1* mutant in the conidia after 24 h incubation on a hydrophobic glass sheet. Among these genes, only three were significantly reduced in transcription (p < 0.01) (S10 Fig). In 70 identified proteins, 13 were associated with oxidoreducation (Fig 7G and S2 Table). Thus, the defect in scavenging host-derived ROS of the Δ*Moyvh1* mutant was associated with the defect in the production of extracellular proteins.

## Discussion

Virulence in the rice blast fungus *M*. *oryzae* is a multifaceted trait contributed by not only the complex circuitry in the pathogen side but also that of the host. In dissecting molecular events leading to virulence, we have previously characterized the dual specificity phosphatase MoYvh1 that shares sequence conservation and functional mechanisms to certain degree with *S*. *cerevisiae* Yvh1. Importantly, we found that MoYvh1 plays a role in not only growth, conidia formation, but also virulence in *M*. *oryzae* [4]. Here, we provided mechanistic evidence to show that MoYvh1 undergoes cytoplasmic to nuclear translocation in response to oxidative stress and that MoYvh1 affects ribosome maturation. Our findings reveal a novel link between ribosome biogenesis and fungal virulence that is mediated by MoYvh1.

In *S*. *cerevisiae*, Yvh1 is a shuttling protein that could remain in the nucleus if fused with a nuclear localization sequence [23,29]. Previous studies also found that Yvh1 binds with the pre-60S ribosome subunit to export it to the cytoplasm. Once arrives there, Yvh1 is released from the pre-ribosome following mature ribosomal protein P0 binding to the ribosomal stalk [23,24,30,31]. How Yvh1 is translocated into the nucleus remains unclear. Through studies of MoYvh1 here, we provided evidence that MoYvh1 exhibits similar nucleo-cytoplasmic shuttling ability and that it functions through interactions with MoSsb1 and MoSsz1.

An unexpected finding is that the interaction between MoYvh1 and MoSsb1 and MoSsz1 differs from aerial hyphae to conidia and the infection stage (Fig 2, S2 and S11 Figs). Similar differentiated interactions were seen before. A BiFC assay showed that Pth11 and Rgs1 interacted *in vivo* during early pathogenesis but not during vegetative growth [32]. The interaction between MoCap1 and MoMac1 was weak during vegetative growth but was enhanced during appressorium formation [33]. In addition, Liu and colleagues showed that MoAtg4 interacts with MoAtg8 only under the nitrogen starvation condition [34]. In view of these findings, we hypothesized that 1) the interaction occurs only under oxidative stress, and 2) MoYvh1 and Hsp70s interactions are developmental stage specific. In agreement with these hypotheses, the YFP fluorescence signal was transferred to the nucleus only following treatment with 5 mM H_2_O_2_ (Fig 2C). Therefore, we concluded that MoSsb1 and MoSsz1 recruitments to MoYvh1 to facilitate its nuclear translocation upon oxidative stress during specific growth stages in *M*. *oryzae*. Further evidence showed that MoYvh1 is not accumulated in the nucleus when MoSsb1 and MoSsz1 are not interacted with MoYvh1 during the aerial hyphae (Fig 1B and S11 Fig). And also, deletion of *MoSSB1* or *MoSSZ1* which interdicted the interaction caused cytoplasmic-MoYvh1 not translocated into the nucleus even in the conidia and infection stages (Fig 2D, S4 and S7 Figs). These results further confirmed that the accumulation of MoYvh1 in the nucleus under oxidative stress is dependent on the interaction between MoSsb1 and MoSsz1. Upon the exposure to oxidative stress, MoYvh1 nuclear translocation is accelerated by its interaction with MoSsb1 or MoSsz1 during the conidial and infection stages. Since MoYvh1 still could be detected in the nucleus in the Δ*Mossb1* and Δ*Mossz1* mutants (S4 Fig), we postulated that additional translocation facilitators of MoYvh1 that are independent of oxidation stress may also exist.

We found that MoYvh1 and MoMrt4 bind with ribosomes in a competitive manner. To further examine the relationship between MoYvh1 and MoMrt4, we characterized the function of MoMrt4 by generating a Δ*Momrt4* mutant, which is significantly attenuated in growth, conidia production, and pathogenicity. However, the lesions produced by the Δ*Momrt4* mutant on rice leaves were fewer and smaller than those of the Δ*Moyvh1* mutant. A DAB assay suggested that deletion of *MoMRT4* did not affect the scavenging of ROS accumulated around the infection sites. Our analysis further suggested that MoYvh1 binds to pre-ribosomes and thereby helps to release MoMrt4. Thus, ribosome immaturity resulted in pathogenicity defects in the Δ*Moyvh1* mutant but not the Δ*Momrt4* mutant. The ribosome extract assay confirmed that cells continue to synthesize ribosomes in the absence of MoMrt4. This finding is consistent with studies in *S*. *cerevisiae* in which the deletion of *MRT4* causes defects in growth but no blocking in ribosome synthesis [5,11,23,35].

*M*. *oryzae* secretes a wide array of factors into the host to facilitate invasion [26,36,37]. However, host plants have evolved to recognize these effectors and counteract by activating defense responses to limit pathogen spreading [38–41]. Small-molecule phytohormones, such as jasmonates, salicylic acid and brassinosteroids, play key roles in regulating this defense response [42–44]. Our recent findings showed that the scavenging of host-derived ROS at the infection site is important for virulence of the Δ*Moyvh1* mutant, as deletion of *MoYVH1* causes virulence defect [4]. Consistent with the findings, the EF of the wild-type scavenges ROS accumulated around the sites of infection, in contrast to the Δ*Moyvh1* mutant, where the restricted invasion is the result of ROS accumulation due to lack of extracellular proteins in EF. When treated with ROS, MoYvh1 in the cytoplasm is translocated into the nucleus causing an enhanced nuclear localization in both the conidia and invasion hyphae (Figs 2, 7C and 7D). In the mycelium, however, the cytoplasmic location pattern of MoYvh1 remained unchanged. These results suggested that the differential localization patterns of MoYvh1 might be developmentally regulated and it may be relevant to virulence. Since H_2_O_2_ stress blocks the formation of appressorium, the glass surface was not subjected to H_2_O_2_ stress allowing conidia to germinate, and under this condition MoYvh1 was present in both nucleus and cytoplasm (S8 Fig). Here, we found that the EF of non-induced Guy11 rescued the defects in invasion and ROS scavenging around the infected sites (Fig 7A and 7B), suggesting that original MoYvh1 in the nucleus (S8 Fig) without treatment regulated the ribosome maturation that provides abundant extracellular proteins to inhibit host-derived ROS. Under ROS stress, MoYvh1 in the cytoplasm translocates into the nucleus and accelerates ribosome synthesis to produce more extracellular proteins, which inhibits host-derived defense and promote infection.

Why does the wild-type EF suppress the defects of the Δ*Moyvh1* mutant and does the EF contain necessary ribosome or other proteins? In *S*. *cerevisiae*, Rpp0 is loaded onto the 60S ribosome subunit to assemble the mature stalk [23,24]. In this study, deletion of *MoYVH1* led to the separation of MoRpp0 from the ribosome, suggesting a ribosomal maturation defect in the Δ*Moyvh1* mutant which would impair the production of proteins (Fig 5D). During the early stages of infection, *M*. *oryzae* secretes various extracellular proteins to suppress plant immunity for promoting colonization. Blockage of secreted protein synthesis due to immature ribosome in the Δ*Moyvh1* mutant likely results in the defect in virulence. Indeed, we showed that EF from the wild type strain restored pathogenicity of the Δ*Moyvh1* mutant when added to the conidia suspension (Fig 7A and 7C and S9 Fig). These findings indicated that MoYvh1 has a role in the production of EFs that inhibit rice immunity.

**Fig 8:**
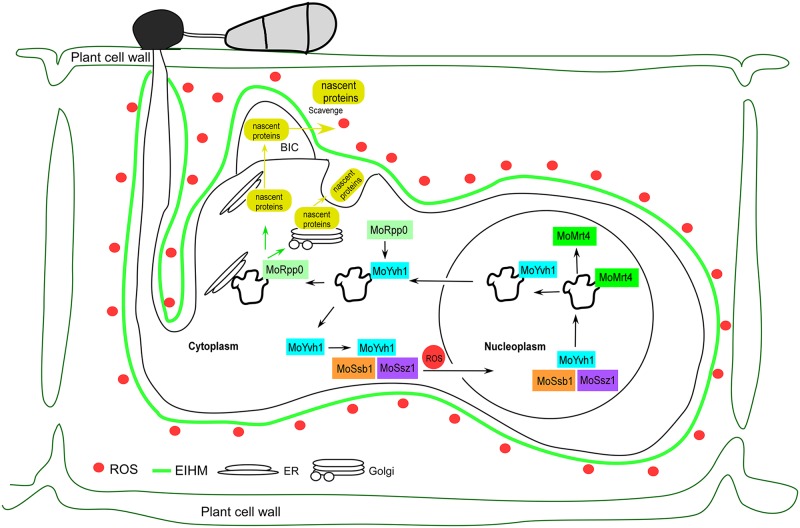
MoYvh1 controls ribosome maturation via the release of MoMrt4 to overcome host innate immunity. A proposed schematic representation of how MoYvh1 desponds to host-derived ROS by nuclear translocation to regulate ribosome maturation through the release of MoMrt4. MoYvh1 and MoMrt4 regulate extracellular protein synthesis that have a role in the evasion of rice innate immunity.

We therefore present a model for how MoYvh1 functions in growth, virulence, and host immune avoidance in *M*. *oryzae* (Fig 8). Our findings demonstrate that during *M*. *oryzae* infection, rice produces an ROS burst to suppress pathogen invasion. Under this stress, MoSsb1 and MoSsz1, together with MoYvh1, are translocated to the nucleus, where MoYvh1 has a role in ribosome maturation through the release of MoMrt4 from the pre-ribosome. Mature ribosomes promote EF synthesis and secretion to scavenge ROS and modulate the rice defense response. Our model reveals an important pathway by which *M*. *oryzae* recruits a nucleocytoplasmic shuttling phosphatase, MoYvh1, in response to host immune response. Further studies of MoYvh1-mediated response and the identification of EFs regulated by MoYvh1 would promote the understanding of *M*. *oryzae* pathogenesis mechanisms.

## Materials and methods

### Strains and culture condition

*M*. *oryzae* Guy11 strain was used as the wild type in this study. All strains were cultured on complete medium (CM) agar plates for 3–15 days at 28°C [45]. Mycelia were harvested from liquid CM and used for DNA and RNA extractions. Protoplasts were prepared and transformed as described previously [46]. Transformants were selected on TB3 medium (3 g of yeast extract, 3 g of casamino acids, 200 g of sucrose, and 7.5 g of agar in 1 l of distilled water) with 300 μg/ml hygromycin B (Roche) or 200 μg/ml zeocin (Invitrogen).

### Ribosomal protein extraction

Ribosomal proteins were extracted from mycelia as previously described [23]. Briefly, fungal strains were cultured on solid CM medium for 7 days at 28°C, and approximately 1 x 1 mm square of agar containing the culture was inoculated in liquid CM and grown for another 2 days. Mycelia were filtered through Miracloth, blotted dry, and ground into powder in liquid nitrogen with a mortar and a pestle. 5 g mycelium was mixed with 15 ml 1^st^ extraction buffer I (0.1 M natrium aceticum, 10 mM Tris-HCl, 10 mM MgCl_2_ with 0.07% β-mercaptoethanol) and incubated at 4°C for 2 h. Samples were centrifuged at 5000 g for 30 min at 4°C and repeated once before discarding the pellets. The upper phase was centrifuged at 96000 g at 4°C for 2 h and the pellet were dissolved in 2^nd^ extraction buffer (20 mM Tris, pH 7.5, 6 mM MgCl_2_, 10% glycerol, 0.1% NP-40, 1 mM PMSF, 1 μM leupeptin, and 1 μM pepstatin A). 2.5 ml of protein extracts were overlaid on 7.5 ml 1 M sucrose in 20 mM Tris, 8 mM MgCl_2_, and 100 mM KCl in 10 ml ultracentrifuge tubes (Beckman Coulter). Samples were centrifuged again at 96000 g at 4°C for 2 h. Finally, the pellets were dissolved in dissolution buffer (5 M Urea, 2 M Thiourea, 2% CHAPS, 2% SB3-10, 40 mM Tris, 5mM Mercaptoethanol).

### Targeted gene disruption and the complementation

To generate the *MoMRT4* gene replacement vector pCX62, approximately 1 kb upstream and downstream fragments were amplified with primer pairs (S1 Table). The resulting PCR products were ligated to the hygromycin resistance cassette released from pCX62, as previously described [25]. Putative mutants were screened by PCR and confirmed by Southern blotting analysis. To complement the Δ*Momrt4* mutant, the DNA fragment containing the putative promoter and the coding sequence was amplified and inserted into pYF11 (bleomycin resistance) by homologous recombination in *S*. *cerevisiae*. Plasmids were extracted and introduced into *Escherichia coli* competent cells, and then the plasmids with correct inserts were introduced into protoplasts, as previously described [25].

### Yeast two-hybrid and BiFC assay

cDNA of *MoYVH1*, *MoYVH1*^ΔC^ (the N-terminus), *MoYVH1*^ΔN^ (the C-terminus), *MoSSB1*, *MoSSZ1* and *MoSSA1* was respectively amplified with Super Fidelity DNA Polymerase (Vazyme, Nanjing). Amplified products were cloned into pGBKT7 and pGADT7 vectors (BD Biosciences, Oxford, UK), respectively. After sequence verification, they were introduced into yeast AH109 strain. Transformants grown on synthetic medium lacking leucine and tryptophan (SD–Leu–Trp) were transferred to synthetic medium lacking leucine, tryptophan and histidine (SD–Leu–Trp–His).

For BiFC assay, the *MoYVH1*-^C^*YFP* fusion construct was generated by cloning *MoYVH1* into pHZ68 [47]. Similarly, *MoSSB1*-^N^*YFP* and *MoSSZ1*-^N^*YFP* fusion constructs were generated by cloning *MoSSB1* and *MoSSZ1* into pHZ65, respectively. Construct pairs of *MoYVH1*-^C^*YFP*, *MoSSB1*-^N^*YFP* and *MoSSZ1*-^N^*YFP* were introduced into the protoplasts of Guy11, respectively. Transformants resistant to both hygromycin and zeocin were isolated and confirmed by PCR.

### Construction of *MoYVH1*^*G69D*^ and *MoYVH1*^*G69E*^

To generate *MoYVH1*^*G69D*^ and *MoYVH1*^*G69E*^ constructs, the 2.7 kb upstream fragment including the *MoYVH1* native promoter, the 1.1 kb fragment from the start codon of the coding sequence (containing the Gly 69), and the 0.5 kb downstream fragment including the rest of the gene coding sequence (containing both of the Gly 69) were co-introduced with *XhoI* digested pYF11 into yeast strain XK1-25 [47,48]. Plasmid pYF11::*MoYVH1*^*G69D*^ and pYF11::*MoYVH1*^*G69E*^ were rescued from the resulting Trp+ yeast transformants.

### Appressorium formation, plant infection, and rice sheath penetration assays

Conidial germination and appressorium formation were measured on a hydrophobic surface as previously described [49]. Appressorium induction and formation rates were obtained also as described previously [50,51].

For infection, conidia were harvested from 10-day-old SDC agar cultures, filtered, and resuspended to a concentration of 5 × 10^4^ spores /ml in a 0.2% (w/v) gelatin solution. For the leaf assay, leaves from two-week-old seedlings of rice (*Oryza sativa* cv. CO39) and 7-day-old seedlings of barley were used for spray inoculation. For rice leaves, 5 ml of a conidial suspension of each treatment was sprayed. Inoculated plants were kept in a growth chamber at 25°C with 90% humidity and in the dark for the first 24 h, followed by a 12 h /12 h light /dark cycle. Lesion formation was observed daily and recorded by photography 7 days after inoculation [52,53].

### HPLC analysis

Mycelia were harvested and ground into powder in liquid nitrogen. 1 mg mycelium was mixed with 20 μl 6% TCA solution, centrifuged (1700 g, 15 min), and top layers were collected. After washing twice with five volumes of anhydrous ether, pellets were collected and subjected to HPLC analysis using a programmable Agilent Technology zorbax 1200 series liquid chromatography. The solvent system consisted of methanol (90%): water (10%), at a flow rate of 1 ml /min. 0.1 mg/ml cAMP solution was eluted through the column (SB-C_18_, 5 μl, 4.6 × 250 mm) and detected at 259 nm UV. Samples were loaded through the column in turns.

### Separation of nuclear proteins and cytoplasm proteins during infection stage

Conidia of indicated strains were harvested from 10-day-old SDC agar cultures, filtered, and resuspended to a concentration of 1 × 10^5^ spores /ml in a 0.2% (w/v) gelatin solution. 4 ml of the suspension was sprayed onto rice leaves and harvested 24 hpi. 5 g of Leaves were ground into powder in liquid nitrogen. The powder was transferred to a 50 ml tube and mixed with 20 ml M1 buffer (10 mM Tris-HCl (pH = 8.0),10 mM MgCl_2_, 0.1 mM PMSF, 1 M NaCl, 0.07% β-mercaptoethanol and 0.4 M Sucrose) using a chilled spoon. After agitated the tube in ice box for 10 min. The suspensions were filtered through Miracloth (Calbiochem) into a 50 ml tube and the supernatants containing cytoplasmic proteins were collected following centrifugation at 1000 x g for 20 min at 4°C. Remove the supernatant for the cytoplasm protein. Five ml of M2 buffer (10 mM Tris-HCl (pH = 8.0), 10 mM MgCl_2_, 0.1 mM PMSF, 1 M NaCl, 0.07% β-mercaptoethanol, 0.25 M Sucrose and 1% TritonX-100) was added to the pellet portion, re-suspended, and tubes re-centrifuged at 12000 x g for 10 min at 4°C. The supernatant was removed and the step was repeated 3 times. Finally, 300 μl Nuclei Lysis Buffer (P0013B, Beyotime Biotech) was added to the pellet and the suspension (nuclear proteins) was recovered.

### Extraction and identification of extracellular fluid proteins

After 7 days cultivation, conidia were collected and suspended in 100 ml of distilled water in a concentration of 1 × 10^5^ spores /ml. 50 ml of the conidia were centrifuged at 3600 g for 10 min to extract the protein for equalization of protein amounts. The rest of 50 ml of conidia were divided into 200 μl (1 x 10^5^ spores/ml) and placed onto the hydrophobic glass sheet at 28°C for 24 h. Suspensions harvested from the hydrophobic glass sheet were centrifuged at 3600 g for 10 min and the supernatants were recovered. For HLPC-MS/MS analysis, a 100 μg protein suspension was harvested. The suspension was mixed with 2.5 μg trypsin for digestion at 37°C for 4 h. 2.5 μg trypsin was added again and incubated for another 8 h. The peptides were then dechlorinated by Strata X and separated by a 65 min gradient elution at a flow rate 300 nl/min with the LC-20AD nano-HPLC system (Shimadzu, Japan), which was directly interfaced with Q-Exactive mass spectrometer (Thermo Fisher Scientific, USA). Mobile phase A consists of 0.1% formic acid and 2% acetonitrile, and mobile phase B consists of 0.1% formic acid and 98% acetonitrile. The mass spectrometer was operated in the DDA (data-dependent acquisition) mode and there was a single full-scan mass spectrum in the Orbitrap (350–1600 m/z, 70,000 resolution).

### Statistical analysis

Results were presented as the mean ± standard deviation (SD) of at least three repeats. The significant differences between samples were statistically evaluated by using SDs and one-way analysis of variance (ANOVA) in SPSS 2.0. The data between two different treatments were then compared statistically by ANOVA, followed by the F-test, if the ANOVA result is significant at *P*< 0.01.

## Supporting information

S1 Fig MoYvh1 translocates into the nucleus in response to KO_2_ and hydroxyl radical. (A) Fluorescence observation of conidia untreated (upper panels) and treated with 1.0 mg/ml KO_2_ and 50mM (-OH) which generated by Fe_2_SO_4_ and H_2_O_2_ for 1 h. Bar = 5 μm. (B) Fluorescence observation of mycelia untreated (upper panels) and treated with 1.0 mg/ml KO_2_ and 50mM (-OH) which generated by Fe_2_SO_4_ and H_2_O_2_ for 1 h. Bar = 5 μm.
(TIF)

S2 Fig MoYvh1 interacts with MoSsb1 and MoSsz1 during infection. (A) BiFC and (B) Co-IP assays for the interaction between MoYvh1 and Hsp70s show that only MoSsb1 and MoSsz1 interact with MoYvh1 during infection. YFP, yellow fluorescent protein.
(TIF)

S3 Fig Yeast two-hybrid analysis of interactions between various domains of MoYvh1 and Hsp70s proteins. MoHsp70s (MoSsa1, MoSsb1 and MoSsz1) cDNA was inserted into the vector pGBKT7 and two truncated parts of MoYvh1 (MoYvh1^ΔC^ and MoYvh1^ΔN^) were inserted into pGADT7. Yeast cells were grown on synthetic dextrose (SD) medium lacking leucine (Leu), tryptophan (Trp), His were investigated with positive and negative controls. Plates were incubated at 30°C for 3 days before being photographed.
(TIF)

S4 Fig Quantitative data of MoYvh1-GFP in Δ*Mossb1*, Δ*Mossz1* and Δ*Moyvh1* mutants during conidia stage. (A) MoYvh1 enriched to the nucleus under oxidative stress in the complement strain. (B) MoYvh1 is not translocated to the nucleus when *MoSSB1* is disrupted. (C) MoYvh1 is not translocated to the nucleus in the Δ*Mossz1*. The intensity of MoYvh1 is compared between the sample treated with/without H_2_O_2_ among total proteins, nuclear proteins, and cytoplasmic proteins. Bars denote standard errors from three independent experiments. Asterisk indicates significant differences (Duncan’s new multiple range test p<0.01).
(TIF)

S5 Fig MoMrt4^G69D^ and MoMrt4^G69E^ suppress the defects in vegetative growth and cAMP levels of the Δ*Moyvh1* mutant. (A) Statistical analyses of the diameter of hypha of wild-type Guy11, the Δ*Moyvh1* mutant, *MoMRT4* and complemented mutant strains. Error bars represent the standard deviations and asterisks denote statistical significances (P<0.01) (B) Bar chart showing quantification of intracellular cAMP in the mycelia stage of all the indicated strains. The error bars represent SD of three replicates. Asterisk indicates significant differences (Duncan’s new multiple range method p<0.01). (C) Statistical analyses of the diameter of hypha of wild type, Δ*Momrt4* and the complement strains. Error bars represent the standard deviations and asterisks denote statistical significances (P<0.01).
(TIF)

S6 Fig Deletion of MoMRT4 did not cause the increased accumulation of ROS of *M*. *oryzae*. DAB assays of Guy11, the Δ*Momrt4* mutant, the complement strains on the rice sheath 30 h after inoculation. Bar = 5 μm.
(TIF)

S7 Fig Localization of MoYvh1 in the Δ*Mossb1* and Δ*Mossz1* mutants during infection. Rice leaves were incubated with (A) Δ*Mssb1/MoYVH1-GFP* and (B) Δ*Mossz1/MoYVH1-GFP* strain for 30 h. Equal weight of rice leaves (LTH and K23) was divided into three parts for extraction of total, nuclear and cytoplasm proteins. Equal amounts of total, nuclear and cytoplasm proteins were separated by SDS-PAGE, and the presence of MoYvh1 was detected by Western blotting using anti-GFP. The intensities of Western blotting bands were quantified with the ODYSSEY infrared imaging system (application software Version 2.1). The intensity of MoYvh1 is compared between the cv. LTH and cv. K23 among total proteins, nuclear proteins, and cytoplasmic proteins. Bars denote standard errors from three independent experiments.
(TIF)

S8 Fig Localization of MoYvh1 during appressorium formation. MoYvh1 was present in both the cytoplasm and the nucleus during appressorium formation. Total proteins were extracted from the conidia and the appressorium of *M*. *oryzae*. The intensity of MoYvh1 is compared between the conidia without H_2_O_2_ treatment (Co) and appressorium (Ap) among total proteins, nuclear proteins, and cytoplasmic proteins. H1 (a nucleus marker) and Actin (a cytoplasm marker) was detected by Western blotting analysis using anti-RFP or anti-Actin antibodies. Bars denote standard errors from three independent experiments. Asterisk indicates significant differences (Duncan’s new multiple range test p<0.01).
(TIF)

S9 Fig The EF of Guy11 recovered the defects of Δ*Moyvh1* in the pathogenicity on the rice cultivar. Conidia of the Δ*Moyvh1* mutant were collected with 5 ml of the EF or boiled EF of Guy11. The conidial suspensions of each treatment were dropped onto the detached rice leaves. “EF” represents the extracellular fluid. “BEF” represents the boiled extracellular fluid.
(TIF)

S10 Fig RT-PCR quantification of selected genes. (A), (B) and (C) RT-PCR quantification of indicated genes. The experiments were repeated three times and showed similar results. Error bars represent the SD and Asterisk indicates significant differences at P<0.01.
(TIF)

S11 Fig MoYvh1 does not interact with MoSsb1 and MoSsz1 during vegetative growth. (A) Co-IP assays for the interaction between MoYvh1 and the Hsp70s. Western blot analysis of total proteins (T) extracted from the mycelium of various transformants, suspension proteins (S) and elution proteins (E), and eluted from anti-GFP beads. The presence of MoYvh1, MoSsa1, MoSsb1 and MoSsz1 was detected with anti-GFP and anti-FLAG antibodies, respectively. (B) BiFC assays for the interaction between MoYvh1 and the Hsp70s showed that MoSsa1, MoSsb1 and MoSsz1 did not interact with MoYvh1 during the vegetative growth stage. YFP, yellow fluorescent protein.
(TIF)

S1 Table Primers used in this study. (DOCX)

S2 Table Putative extracellular proteins which lack in the EFs of Δ*Moyvh1* mutants. (DOCX)
