# Supplementary figures and images for: MoYvh1 subverts rice defense through functions of ribosomal protein MoMrt4 in Magnaporthe oryzae
Source: PLoS Pathog. 2018 Apr 23;14(4):e1007016. doi: 10.1371/journal.ppat.1007016 (PMC5933821; doi:10.1371/journal.ppat.1007016)

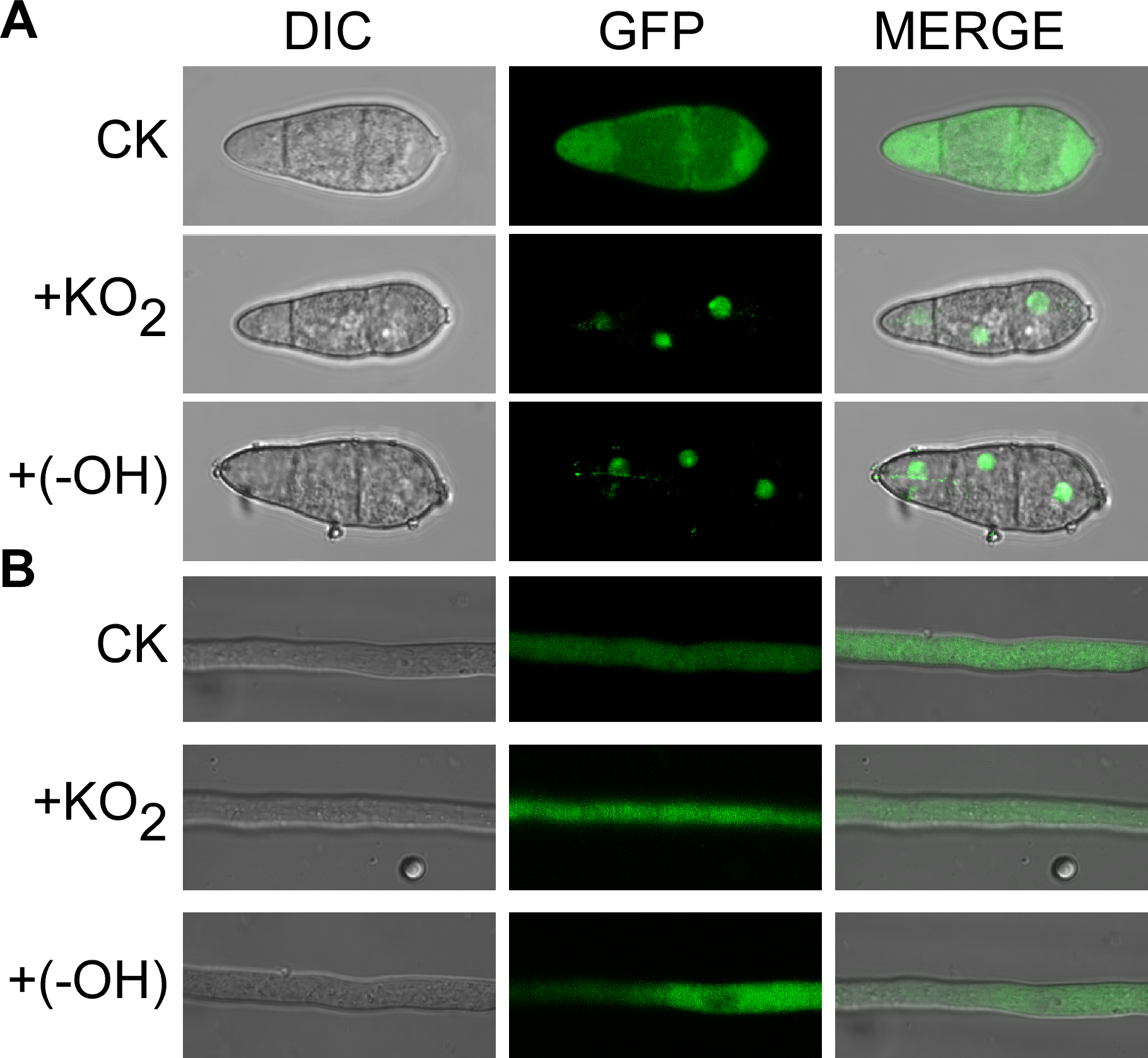

Supplement: S1 Fig — (A) Fluorescence observation of conidia untreated (upper panels) and treated with 1.0 mg/ml KO2 and 50mM (-OH) which generated by Fe2SO4 and H2O2 for 1 h. Bar = 5 μm. (B) Fluorescence observation of mycelia untreated (upper panels) and treated with 1.0 mg/ml KO2 and 50mM (-OH) which generated by Fe2SO4 and H2O2 for 1 h. Bar = 5 μm. (TIF) [file ppat.1007016.s001.tif]

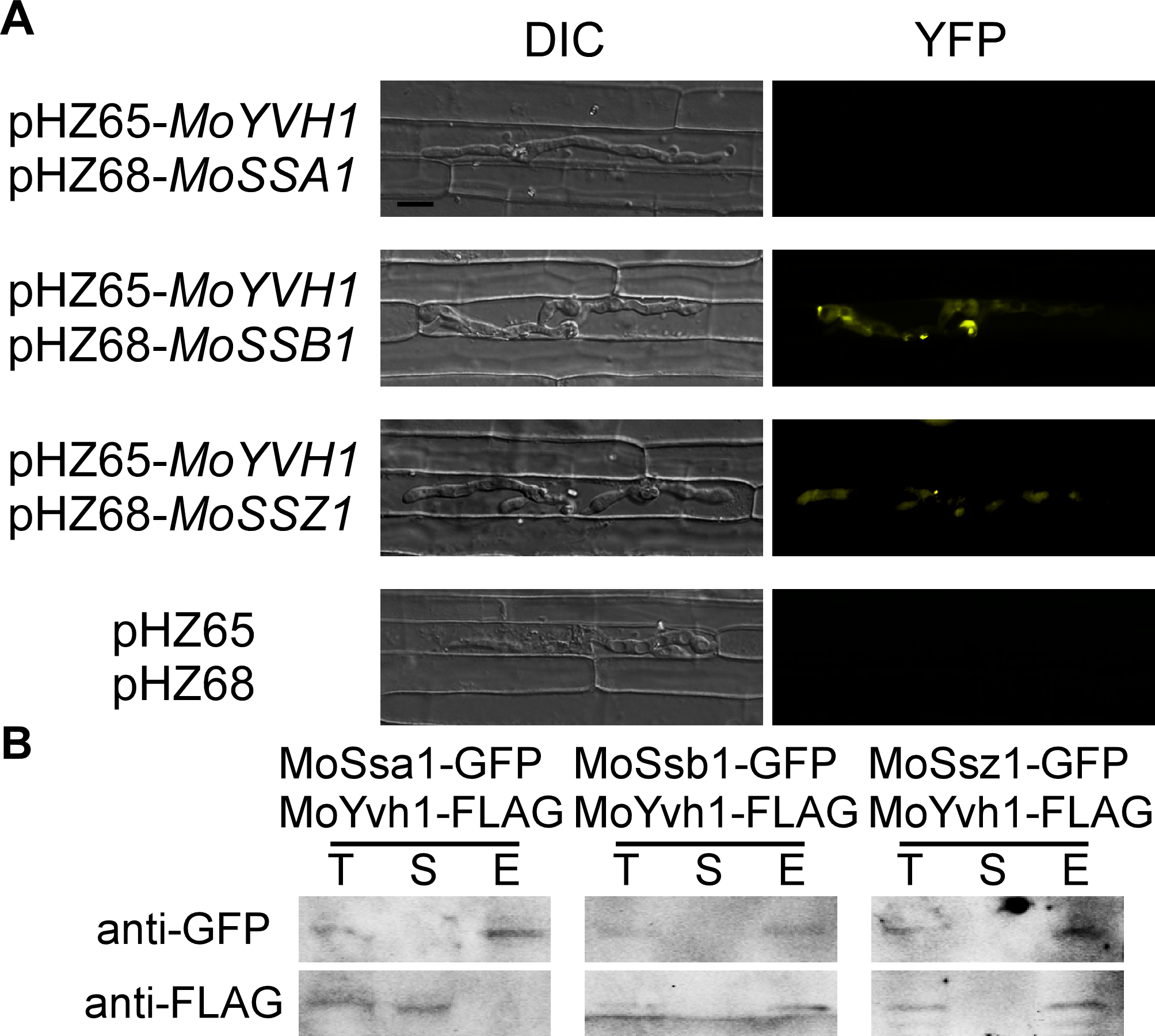

Supplement: S2 Fig — (A) BiFC and (B) Co-IP assays for the interaction between MoYvh1 and Hsp70s show that only MoSsb1 and MoSsz1 interact with MoYvh1 during infection. YFP, yellow fluorescent protein. (TIF) [file ppat.1007016.s002.tif]

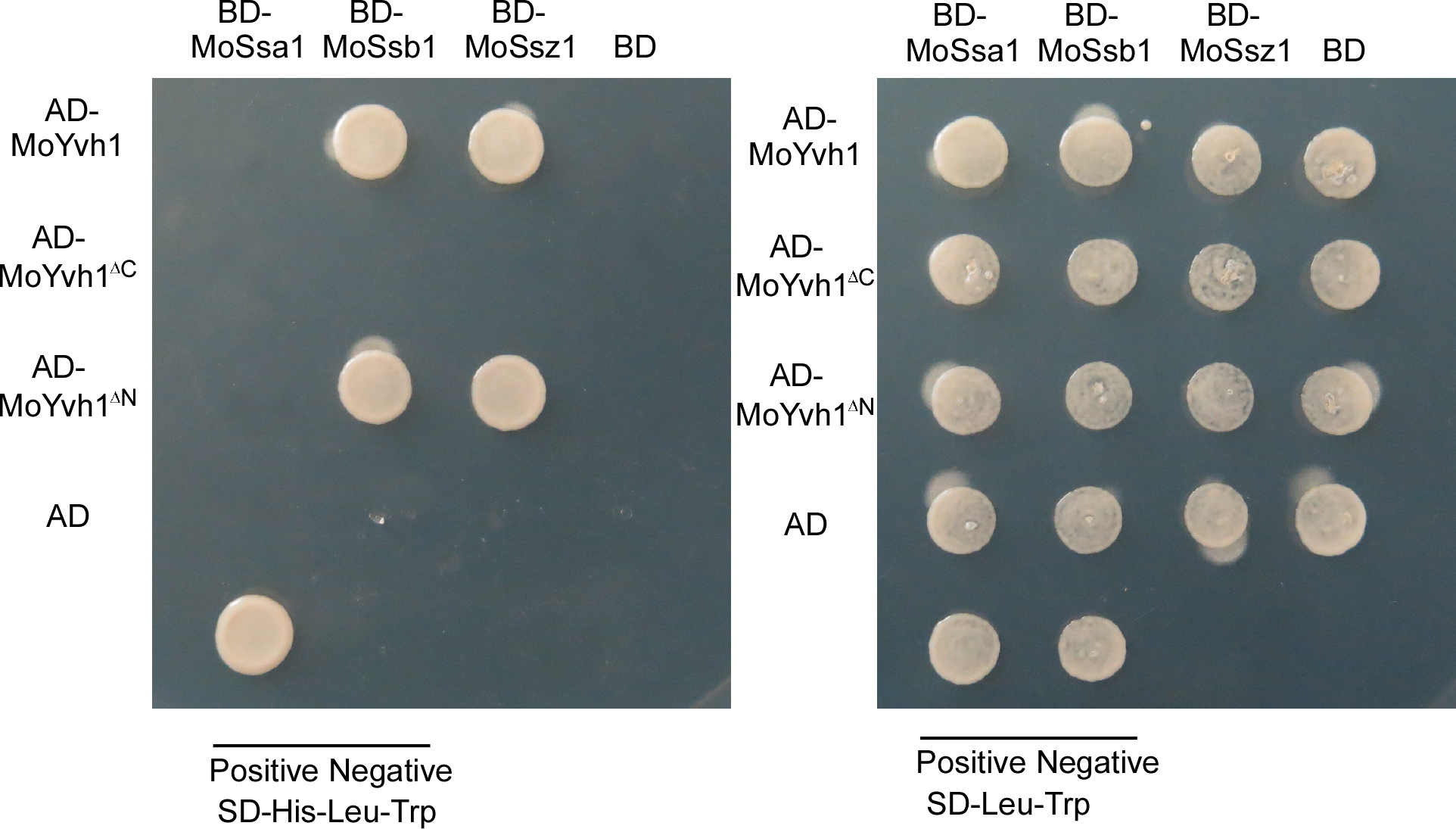

Supplement: S3 Fig — MoHsp70s (MoSsa1, MoSsb1 and MoSsz1) cDNA was inserted into the vector pGBKT7 and two truncated parts of MoYvh1 (MoYvh1ΔC and MoYvh1ΔN) were inserted into pGADT7. Yeast cells were grown on synthetic dextrose (SD) medium lacking leucine (Leu), tryptophan (Trp), His were investigated with positive and negative controls. Plates were incubated at 30°C for 3 days before being photographed. (TIF) [file ppat.1007016.s003.tif]

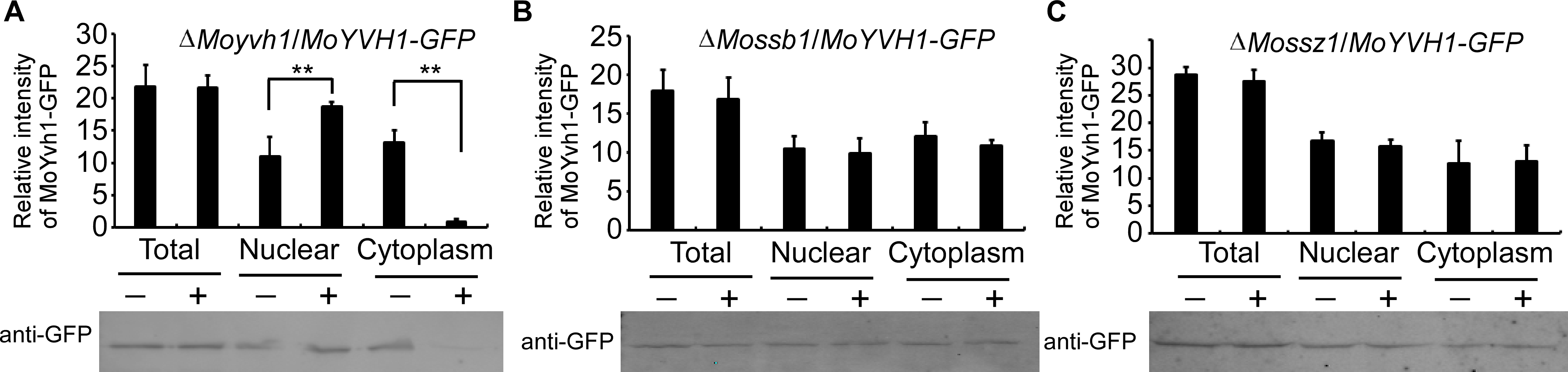

Supplement: S4 Fig — (A) MoYvh1 enriched to the nucleus under oxidative stress in the complement strain. (B) MoYvh1 is not translocated to the nucleus when MoSSB1 is disrupted. (C) MoYvh1 is not translocated to the nucleus in the ΔMossz1. The intensity of MoYvh1 is compared between the sample treated with/without H2O2 among total proteins, nuclear proteins, and cytoplasmic proteins. Bars denote standard errors from three independent experiments. Asterisk indicates significant differences (Duncan’s new multiple range test p<0.01). (TIF) [file ppat.1007016.s004.tif]

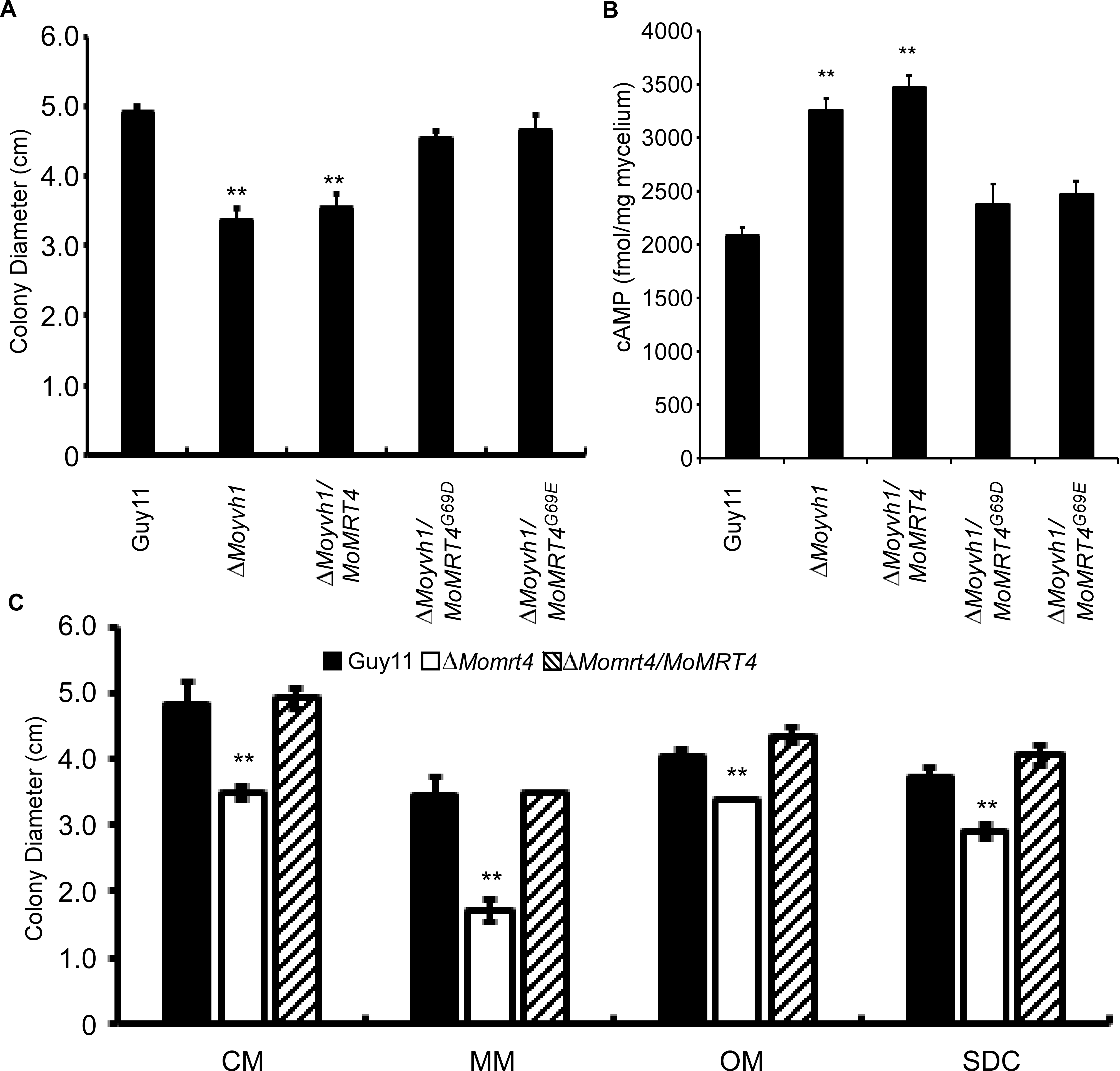

Supplement: S5 Fig — (A) Statistical analyses of the diameter of hypha of wild-type Guy11, the ΔMoyvh1 mutant, MoMRT4 and complemented mutant strains. Error bars represent the standard deviations and asterisks denote statistical significances (P<0.01) (B) Bar chart showing quantification of intracellular cAMP in the mycelia stage of all the indicated strains. The error bars represent SD of three replicates. Asterisk indicates significant differences (Duncan’s new multiple range method p<0.01). (C) Statistical analyses of the diameter of hypha of wild type, ΔMomrt4 and the complement strains. Error bars represent the standard deviations and asterisks denote statistical significances (P<0.01). (TIF) [file ppat.1007016.s005.tif]

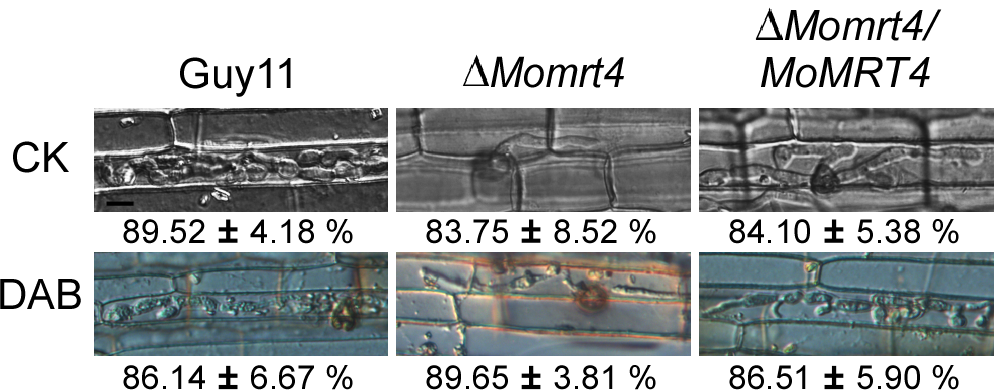

Supplement: S6 Fig — DAB assays of Guy11, the ΔMomrt4 mutant, the complement strains on the rice sheath 30 h after inoculation. Bar = 5 μm. (TIF) [file ppat.1007016.s006.tif]

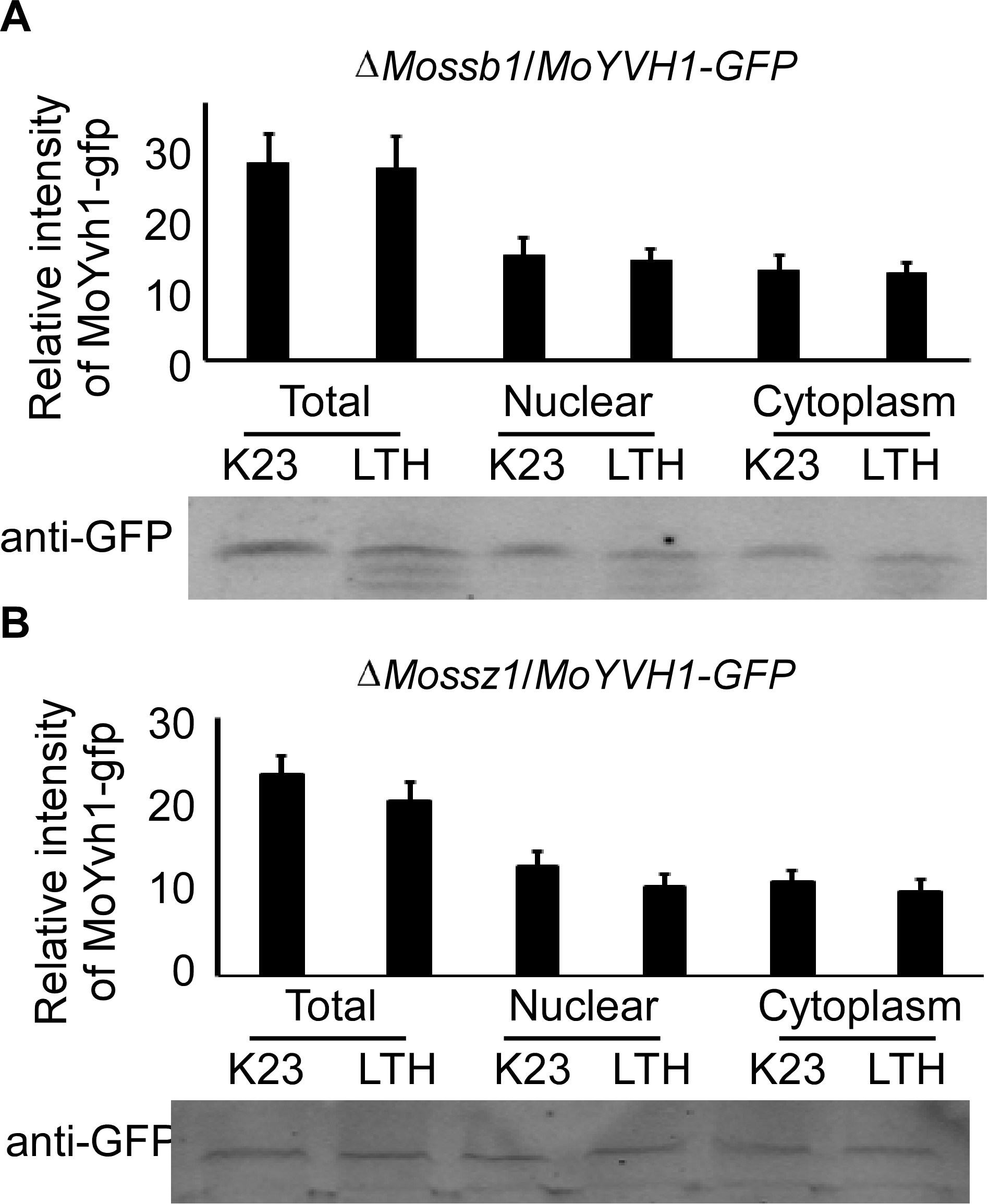

Supplement: S7 Fig — Rice leaves were incubated with (A) ΔMssb1/MoYVH1-GFP and (B) ΔMossz1/MoYVH1-GFP strain for 30 h. Equal weight of rice leaves (LTH and K23) was divided into three parts for extraction of total, nuclear and cytoplasm proteins. Equal amounts of total, nuclear and cytoplasm proteins were separated by SDS-PAGE, and the presence of MoYvh1 was detected by Western blotting using anti-GFP. The intensities of Western blotting bands were quantified with the ODYSSEY infrared imaging system (application software Version 2.1). The intensity of MoYvh1 is compared between the cv. LTH and cv. K23 among total proteins, nuclear proteins, and cytoplasmic proteins. Bars denote standard errors from three independent experiments. (TIF) [file ppat.1007016.s007.tif]

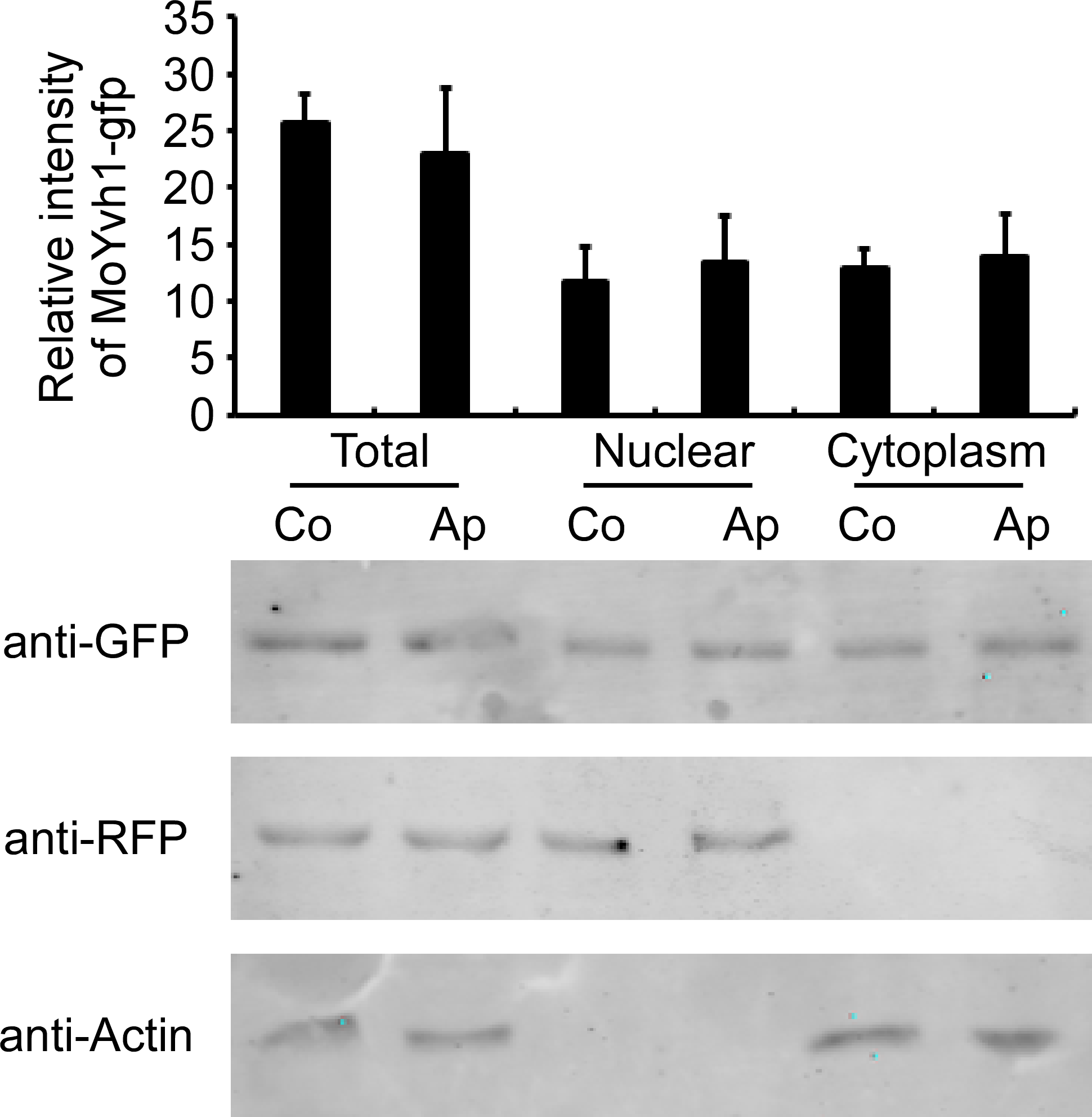

Supplement: S8 Fig — MoYvh1 was present in both the cytoplasm and the nucleus during appressorium formation. Total proteins were extracted from the conidia and the appressorium of M. oryzae. The intensity of MoYvh1 is compared between the conidia without H2O2 treatment (Co) and appressorium (Ap) among total proteins, nuclear proteins, and cytoplasmic proteins. H1 (a nucleus marker) and Actin (a cytoplasm marker) was detected by Western blotting analysis using anti-RFP or anti-Actin antibodies. Bars denote standard errors from three independent experiments. Asterisk indicates significant differences (Duncan’s new multiple range test p<0.01). (TIF) [file ppat.1007016.s008.tif]

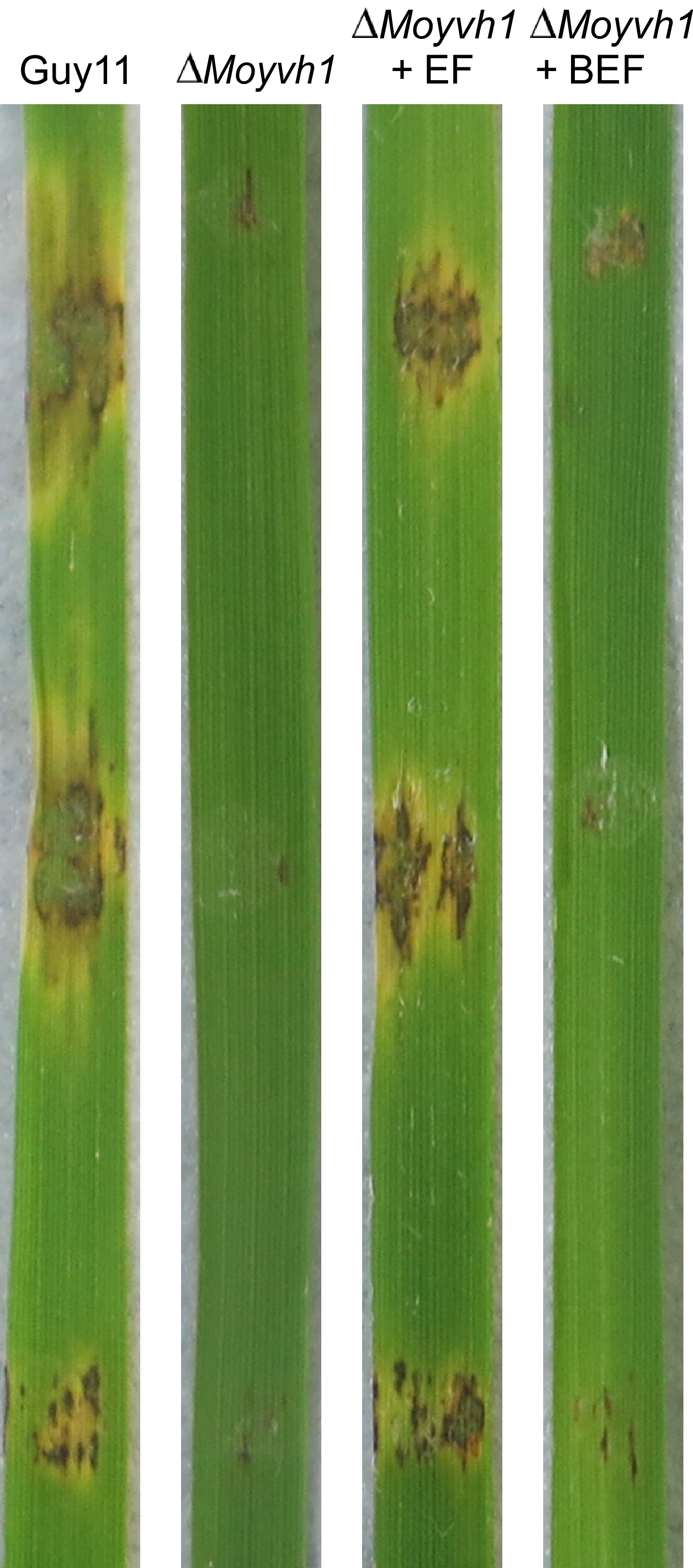

Supplement: S9 Fig — Conidia of the ΔMoyvh1 mutant were collected with 5 ml of the EF or boiled EF of Guy11. The conidial suspensions of each treatment were dropped onto the detached rice leaves. “EF” represents the extracellular fluid. “BEF” represents the boiled extracellular fluid. (TIF) [file ppat.1007016.s009.tif]

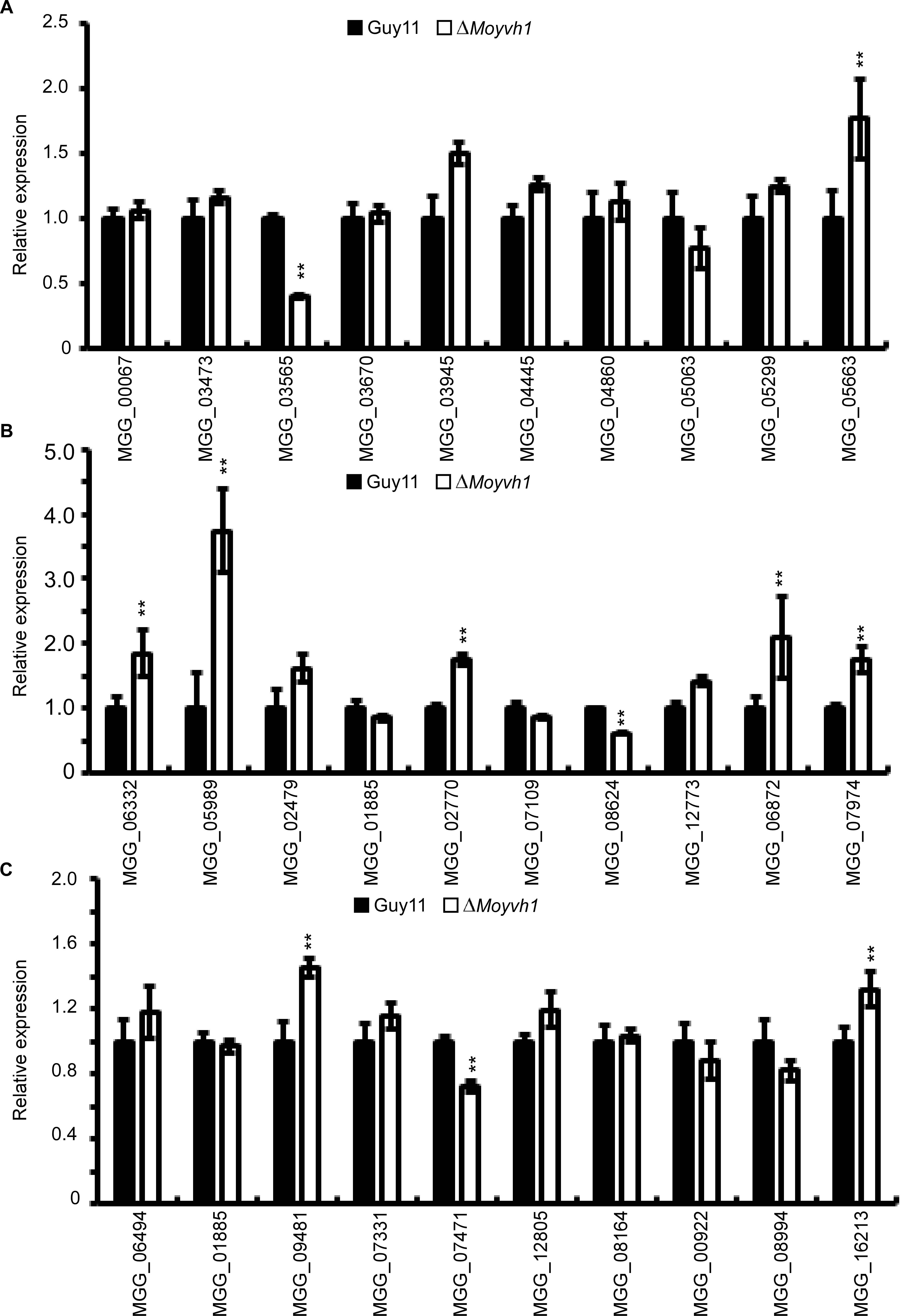

Supplement: S10 Fig — (A), (B) and (C) RT-PCR quantification of indicated genes. The experiments were repeated three times and showed similar results. Error bars represent the SD and Asterisk indicates significant differences at P<0.01. (TIF) [file ppat.1007016.s010.tif]

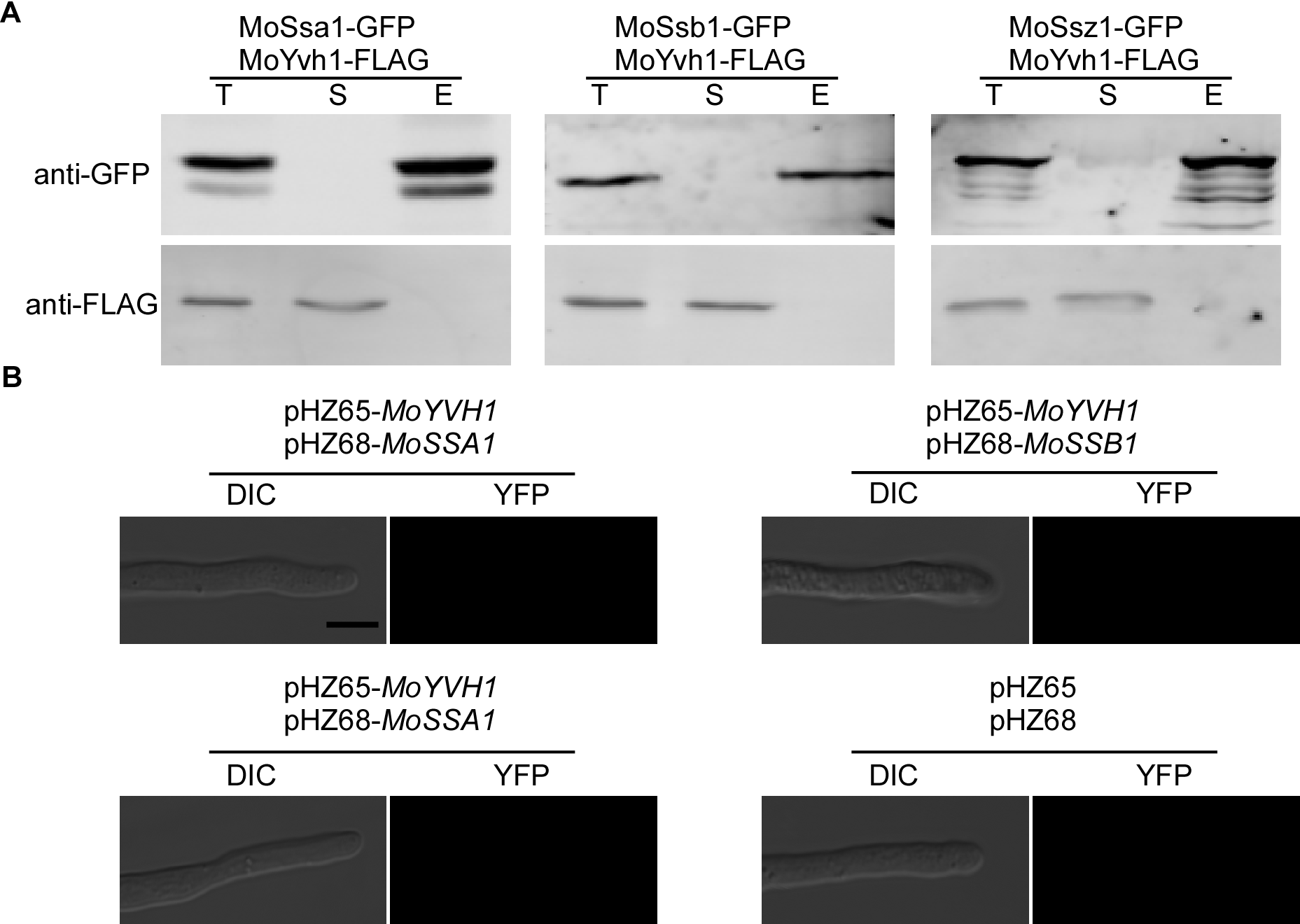

Supplement: S11 Fig — (A) Co-IP assays for the interaction between MoYvh1 and the Hsp70s. Western blot analysis of total proteins (T) extracted from the mycelium of various transformants, suspension proteins (S) and elution proteins (E), and eluted from anti-GFP beads. The presence of MoYvh1, MoSsa1, MoSsb1 and MoSsz1 was detected with anti-GFP and anti-FLAG antibodies, respectively. (B) BiFC assays for the interaction between MoYvh1 and the Hsp70s showed that MoSsa1, MoSsb1 and MoSsz1 did not interact with MoYvh1 during the vegetative growth stage. YFP, yellow fluorescent protein. (TIF) [file ppat.1007016.s011.tif]
