# Supplementary material for: MoYvh1 subverts rice defense through functions of ribosomal protein MoMrt4 in Magnaporthe oryzae
Source: PLoS Pathog. 2018 Apr 23;14(4):e1007016. doi: 10.1371/journal.ppat.1007016 (PMC5933821; doi:10.1371/journal.ppat.1007016)
Supplement: S1 Table — (DOCX) [file ppat.1007016.s012.docx]

**Table S1 Primers used in this study**

| **Primer name** | **Sequence (5’-3’)** | **Remark** |
| --- | --- | --- |
| MGG_08908koF1 | TAACTCGAGGTTCCAGACTCTAGAGCTAGTC | amplify *MoMRT4* 5’ flank sequence |
| MGG_08908koF2 | TAAGAATTCCGCGACTTGTCTGTGAGGCTGT | amplify *MoMRT41* 5’ flank sequence |
| MGG_08908koF3 | TAAACTAGTGTTCGATCCGGTCCGAGTACGT | amplify *MoMRT4* 3’ flank sequence |
| MGG_08908koF4 | TAAGAGCTCCATGTACTGGTACAGCACAGCC | amplify *MoMRT4* 3’ flank sequence |
| MGG_08908 F | ACTCACTATAGGGCGAATTGGGTACTCAAATTGGTTTCTCGTCATGCAGATGCTCCTC | Construction of *MoMRT4-GFP* |
| MGG_08908 R | CACCACCCCGGTGAACAGCTCCTCGCCCTTGCTCACGTCGCTATCGTCTATGGCATCA | Construction of *MoMRT4-GFP* |
| MGG_08908 conF | GTGGTCCACATGACCAACGTGG | validation of *Momrt4* deletion |
| MGG_08908 conR | AAGTCGACCGACGTGATCGAGT | validation of *Momrt4* deletion |
| MGG_08908 BY | CCAACGAAGTGTGTAACGGGTT | validation of *Momrt4* deletion (*HPH*) |
| MGG_09700 F | ACTCACTATAGGGCGAATTGGGTACTCAAATTGGTTGCAGTGAACTTGTGTAGGCACC | Construction of *MoYVH1-GFP* |
| MGG_09700R | CACCACCCCGGTGAACAGCTCCTCGCCCTTGCTCACGAGATTTTCCCTCAAGGACGGC | Construction of *MoYVH1-GFP* |
| AD-MoYvh1 F1 | GTACCAGATTACGCTCATATGATGGCCTTGTCACGCATTAATG | Construction of AD-MoYvh1 |
| AD-MoYvh1 F2 | ATGCCCACCCGGGTGGAATTCTCAGAGATTTTCCCTCAAGGAC | Construction of AD-MoYvh1 |
| BD-MoSsb1 F1 | TCAGAGGAGGACCTGCATATGATGTCGACAGAAGTTTATGACG | Construction of BD-MoSsb1 |
| BD-MoSsb1 F2 | TCGACGGATCCCCGGGAATTCTTAGCGGGAAGACATGGCCTTG | Construction of BD-MoSsb1 |
| BD-MoSsz1 F1 | TCAGAGGAGGACCTGCATATGATGAGCGAAAACGGCACAAAGG | Construction of BD-MoSsz1 |
| BD-MoSsz1 F2 | TCGACGGATCCCCGGGAATTCTCACAATGTACCCCTGACACCA | Construction of BD-MoSsz1 |
| BD-MoSsa1 F1 | TCAGAGGAGGACCTGCATATGATGGCGCCCGCTGTTGGTATCG | Construction of BD-MoSsa1 |
| BD-MoSsa1 F2 | TCGACGGATCCCCGGGAATTCTTAGTCGACCTCCTCGACAGTG | Construction of BD-MoSsa1 |
| AD-MoYvh1*^ΔC^* F1 | GTACCAGATTACGCTCATATGATGGCCTTGTCACGCATTAATG | Construction of AD-MoYvh1*^ΔC^* |
| AD-MoYvh1*^ΔC^* F2 | ATGCCCACCCGGGTGGAATTCTCACTTGGCCGACTCCTCGTCCTCG | Construction of AD-MoYvh1*^ΔC^* |
| AD-MoYvh1*^ΔN^* F1 | GTACCAGATTACGCTCATATGATGGAGGAGGATGCTGCTGCCA | Construction of AD-MoYvh1*^ΔN^* |
| AD-MoYvh1*^ΔN^* F2 | ATGCCCACCCGGGTGGAATTCTCAGAGATTTTCCCTCAAGGACGGC | Construction of AD-MoYvh1*^ΔN^* |
| MGG_09700nls F1 | TTTCGTAGGAACCCAATCTTCAAAATGGAGGAGGATGCTGCTGCCA | Construction of *MoYVH1-nls-GFP* |
| MGG_09700nls F2 | ATGCGTGACAAGGCCATAACCTTTCTCTTCTTCTTAGGAACCTTTCTCTTCTTCTTAGGAACCTTTCTCTTCTTCTTAGGCTTGTACAGCTCGTCCATG | Construction of *MoYVH1-nls-GFP* |
| MGG_09700nls F3 | CATGGACGAGCTGTACAAGCCTAAGAAGAAGAGAAAGGTTCCTAAGAAGAAGAGAAAGGTTCCTAAGAAGAAGAGAAAGGTTATGGCCTTGTCACGCAT | Construction of *MoYVH1-nls-GFP* |
| MGG_09700nls F4 | GCGCAGAGGAGCGTGAATGTTGAGTGGAATGATGAGATTTTCCCTCAAGGACGG | Construction of *MoYVH1-nls-GFP* |
| MGG_08908G69D F1 | ACTCACTATAGGGCGAATTGGGTACTCAAATTGGTTTCTCGTCATGCAGATGCTCCTC | Construction of *MoMRT4^G69D^* |
| MGG_08908G69D F2 | CGGGCCATGAGTTTGGTCTTGTCGAAGAACATGCGGCTATCACC | Construction of *MoMRT4^G69D^* |
| MGG_08908G69D F3 | GGTGATAGCCGCATGTTCTTCGACAAGACCAAACTCATGGCCCG | Construction of *MoMRT4^G69D^* |
| MGG_08908G69D F4 | CACCACCCCGGTGAACAGCTCCTCGCCCTTGCTCACGTCGCTATCGTCTATGGCATCA | Construction of *MoMRT4^G69D^* |
| MGG_08908G69E F1 | ACTCACTATAGGGCGAATTGGGTACTCAAATTGGTTTCTCGTCATGCAGATGCTCCTC | Construction of *MoMRT4^G69E^* |
| MGG_08908G69E F2 | CGGGCCATGAGTTTGGTCTTCTCGAAGAACATGCGGCTATCACC | Construction of *MoMRT4^G69E^* |
| MGG_08908G69E F3 | GGTGATAGCCGCATGTTCTTCGAGAAGACCAAACTCATGGCCCG | Construction of *MoMRT4^G69E^* |
| MGG_08908G69E F4 | CACCACCCCGGTGAACAGCTCCTCGCCCTTGCTCACGTCGCTATCGTCTATGGCATCA | Construction of *MoMRT4^G69E^* |
| pHZ65-09700F1 | CGACTCACTATAGGGCGAATTGGGTACTCAAATTGGCAGTGAACTTGTGTAGGCACC | Construction of pHZ65-*MoYVH1* |
| pHZ65-09700F2 | GCTCACCATCGTGGCGATGGAGCGGAGATTTTCCCTCAAGGACGGC | Construction of pHZ65-*MoYVH1* |
| pHZ68-MoSsb1F1 | CGACTCACTATAGGGCGAATTGGGTACTCAAATTGGCACCTGCCTTGGGCTACGGAG | Construction of pHZ68-*MoSSB1* |
| pHZ68-MoSsb1F2 | GTTCGGGATCTTGCAGGCCGGGCGGCGGGAAGACATGGCCTTGGTG | Construction of pHZ68-*MoSSB1* |
| pHZ68-MoSsz1F1 | CGACTCACTATAGGGCGAATTGGGTACTCAAATTGGTCGATCAAGAAGATACAGGCC | Construction of pHZ68-*MoSSZ1* |
| pHZ68-MoSsz1F2 | GTTCGGGATCTTGCAGGCCGGGCGCAATGTACCCCTGACACCACCC | Construction of pHZ68-*MoSSZ1* |
| pHZ68-MoSsa1F1 | CGACTCACTATAGGGCGAATTGGGTACTCAAATTGAACACCAGTGCTCTGATCGATG | Construction of pHZ68-*MoSSA1* |
| pHZ68-MoSsa1F2 | GTTCGGGATCTTGCAGGCCGGGCGGTCGACCTCCTCGACAGTGGGG | Construction of pHZ68-*MoSSA1* |
| MGG_09700-FLAG F1 | CTATAGGGCGAATTGGGTACTCAAATTGGTTGCAGTGAACTTGTGTAGGCACC | Construction of *MoYVH1-FLAG* |
| MGG_09700-FLAG F2 | CTTTATAATCACCGTCATGGTCTTTGTAGTCGAGATTTTCCCTCAAGGACGGC | Construction of *MoYVH1-FLAG* |
| MGG_04467-FLAG F1 | CTATAGGGCGAATTGGGTACTCAAATTGGTTGAGTGCAGTCAGCATCGAGACA | Construction of *MoRpp0-FLAG* |
| MGG_04467-FLAG F2 | CTTTATAATCACCGTCATGGTCTTTGTAGTCGTCGAAGAGACCGAAGCCCATG | Construction of *MoRpp0-FLAG* |
| MGG_00067 F1 | CCATGGTGTCTGACGTGGCTG | qRT for the genes absent in EF |
| MGG_00067 F2 | CATGATGTGATGACATCAGC | qRT for the genes absent in EF |
| MGG_03473 F1 | GAGGGTCAATGGAAGAAGACG | qRT for the genes absent in EF |
| MGG_03473 F2 | AGGCTGCAGGCCAAGCAGATCC | qRT for the genes absent in EF |
| MGG_03565 F1 | CGAGGCGTACGAGGTCCTCAG | qRT for the genes absent in EF |
| MGG_03565 F2 | GGAACATGAACTGCTGGCCG | qRT for the genes absent in EF |
| MGG_03670 F1 | CCGTCGGTATTCTTACCGATATCC | qRT for the genes absent in EF |
| MGG_03670 F2 | GCTCGATAGCCTTCTCAAGCTC | qRT for the genes absent in EF |
| MGG_03945 F1 | GCGTCGCTTGCACAGGAGCCTCC | qRT for the genes absent in EF |
| MGG_03945 F2 | GACCATGTCGTAGCTTTGATCG | qRT for the genes absent in EF |
| MGG_04445 F1 | TCCACATCTACAAGTCTACCG | qRT for the genes absent in EF |
| MGG_04445 F2 | CGAGGTACGTGTTGGCCTGGAC | qRT for the genes absent in EF |
| MGG_04860 F1 | CAGAGGCAGTAGATGCTGACGC | qRT for the genes absent in EF |
| MGG_04860 F2 | CGAGTCTGGGTGATGACATC | qRT for the genes absent in EF |
| MGG_05063 F1 | GTGTCGAGCTGTTCCAGAGCG | qRT for the genes absent in EF |
| MGG_05063 F2 | CGCAGCCGCTGACGGCAGCGTC | qRT for the genes absent in EF |
| MGG_05299 F1 | CAGCCTCTGTCGATGGTCGCATC | qRT for the genes absent in EF |
| MGG_05299 F2 | CGTTGACATCAACAGCCGAATGC | qRT for the genes absent in EF |
| MGG_05663 F1 | GACTGGATGCAGCCATACCACC | qRT for the genes absent in EF |
| MGG_05663 F2 | GTTGTAGTCCTTCTTGCCCTTC | qRT for the genes absent in EF |
| MGG_06332 F1 | GATGACCAAGGAGAGAGTGG | qRT for the genes absent in EF |
| MGG_06332 F2 | CCTTGCTTGCCGCACCACTGGC | qRT for the genes absent in EF |
| MGG_05989 F1 | CCATGATCGACCTCCTGGTCG | qRT for the genes absent in EF |
| MGG_05989 F2 | GGCCCTCACGCTGCTTCTCAG | qRT for the genes absent in EF |
| MGG_02479 F1 | GAAGCTGTCGGGCTTGCCTTTCG | qRT for the genes absent in EF |
| MGG_02479 F2 | GCCTTGAGAAGCTGGAATACC | qRT for the genes absent in EF |
| MGG_01885 F1 | GGTACGAGTTCGGCTTCGGCCTG | qRT for the genes absent in EF |
| MGG_01885 F2 | CCACGTCGAACAGGTCCACG | qRT for the genes absent in EF |
| MGG_02770 F1 | CATTGTCAAGAGCGCAATTG | qRT for the genes absent in EF |
| MGG_02770 F2 | CCAGAATAGCAACACCGCCAG | qRT for the genes absent in EF |
| MGG_07109 F1 | GCTGCGTGTCACCAACGTCTCG | qRT for the genes absent in EF |
| MGG_07109 F2 | GCCTTGACGGCATCTTCTCTG | qRT for the genes absent in EF |
| MGG_08624 F1 | CGACCAAGTACATCAGCGTTC | qRT for the genes absent in EF |
| MGG_08624 F2 | CAGGATGTTGAAGTAGTCGTCG | qRT for the genes absent in EF |
| MGG_12773 F1 | GGAGAAGATCGAGGAGCTGCTGG | qRT for the genes absent in EF |
| MGG_12773 F2 | CAGGCCTGGTGGTAGCAGTCGTCC | qRT for the genes absent in EF |
| MGG_06872 F1 | GGCTGGAGGTTCGAGAACTGC | qRT for the genes absent in EF |
| MGG_06872 F2 | GGACGTCACTCTCCCCTGCCTC | qRT for the genes absent in EF |
| MGG_07974 F1 | GCTATCCAACGACCTTTCAGAC | qRT for the genes absent in EF |
| MGG_07974 F2 | GTTGATCAGGACGTCGATCTTGG | qRT for the genes absent in EF |
| MGG_06494 F1 | CCATGTCGGGCAGCATCGTCAAC | qRT for the genes absent in EF |
| MGG_06494 F2 | CCATGTACCCAGGGGAGATGCAG | qRT for the genes absent in EF |
| MGG_01885 F1 | GGTACGAGTTCGGCTTCGGCCTG | qRT for the genes absent in EF |
| MGG_01885 F2 | CCACGTCGAACAGGTCCACG | qRT for the genes absent in EF |
| MGG_09481 F1 | GCTCGTCTTCCTCGGCAGCG | qRT for the genes absent in EF |
| MGG_09481 F2 | CTGATAGGCCGTTGAGTCCTGG | qRT for the genes absent in EF |
| MGG_07331 F1 | GATCGATGGCGACAAAGTTG | qRT for the genes absent in EF |
| MGG_07331 F2 | CGGTTCCGATAAGCCAGTGC | qRT for the genes absent in EF |
| MGG_07471 F1 | GAGATTGCTGTTGAGAACGAAG | qRT for the genes absent in EF |
| MGG_07471 F2 | CTCCTAACAGTGGCAAAGTTGC | qRT for the genes absent in EF |
| MGG_12805 F1 | CGTCAAGCTGCTGGGAGTTGAG | qRT for the genes absent in EF |
| MGG_12805 F2 | CCAGCCGACACCGAGTGGGTGTCG | qRT for the genes absent in EF |
| MGG_08164 F1 | CCAGGGCGTCCAGTCTTACCCC | qRT for the genes absent in EF |
| MGG_08164 F2 | GTGCCCGAGGTGGCATCAACACC | qRT for the genes absent in EF |
| MGG_00922 F1 | TGCTGAGCTCGACAACACCG | qRT for the genes absent in EF |
| MGG_00922 F2 | CCAGGATGTAGTCATAGGCGCT | qRT for the genes absent in EF |
| MGG_08994 F1 | CGTGACGGACGTGTGCACCACC | qRT for the genes absent in EF |
| MGG_08994 F2 | GACGATGCTGAAGAGCGAGACC | qRT for the genes absent in EF |
