# Supplementary material for: MoYvh1 subverts rice defense through functions of ribosomal protein MoMrt4 in Magnaporthe oryzae
Source: PLoS Pathog. 2018 Apr 23;14(4):e1007016. doi: 10.1371/journal.ppat.1007016 (PMC5933821; doi:10.1371/journal.ppat.1007016)
Supplement: S2 Table — (DOCX) [file ppat.1007016.s013.docx]

| **Gene ID** | **Predicted Function** | **Number of Peptides** | **Sequence of Peptides** |
| --- | --- | --- | --- |
|  | **Oxidoreduction** |  |  |
| MGG_00067 | glycerol-3-phosphate dehydrogenase [NAD+] activity | 4 | DSSVLVFNLPHEFLGK;EGDYPLFTTVNEILEGK;GVSVAEIEEK;LPSNIIANPSLTDAVR |
| MGG_00175 | phosphogluconate dehydrogenase (decarboxylating) activity | 21 | AINNAQPGWR;DIFQSISAK;DIMYFNDEDGLPLVEK;DITSAYR;DLEQALYASK;DLPANLLQAQR;DYFGAHTFR;EIGDVFTQWNK;GDIIIDGGNSFFPDSNR;GLQFVGSGVSGGEEGAR;GVLDSFLIEITR;IISYAQGFMLMQEAAK;IMLLVQAGK;KNPDLENLLFDDFFNK;LEYIGR;LNKPSIALMWR;NPDLENLLFDDFFNK;SIVGAHSTEEFVSK;TSSFEGNKEQFIK;WTTINALDLGMPVTLIGEAVFGR;YGPSIMPGGSEAAWPHIK |
| MGG_00994 | coproporphyrinogen oxidase activity | 1 | LGDLTGNPVFTELALK |
| MGG_01061 | coproporphyrinogen oxidase activity | 1 | VESILMSLPR |
| MGG_01387 | exonuclease activity | 2 | IPDGITVFR;SEGNAVLVFGDTNSR |
| MGG_03483 | NADH dehydrogenase (quinone) activity | 1 | YFLLSEMGR |
| MGG_03619 | succinate dehydrogenase activity | 7 | ASNTLDFHTR;ATSGINGALTR;FPGMAITYALMQR;LGQISLHIDPTQPGK;TFTTYNAIAEGK;TQVEHGIQDSVK;VAGDTASNYLFR |
| MGG_04337 | catalase activity | 14 | ADLVFGSHSELR;AILATGVAPSK;AQQLTLTAPELTVLVGGLR;FAPLNSWPDNVSLDK;FTMNYLEYLFK;GDATQEQTDVESFTHLEPAADAFR;GPDTITSGLEVIWTK;LIQTAWASASTFR;MLTTDLSLR;NAEEFIPDAFDPSK;SLDYEGLK;VTTEQIMVDR;WEVNNPQQLAEVLK;YEWELTK |
| MGG_04689 | hydrolase activity, hydrolyzing O-glycosyl compounds | 6 | ADFLQEFTTAQNLEGAPGTFNAVR;AGVGAQPDQLVAFIGDFK;AVLDAIDWVGVDEYPYYENGK;ILLGVWTSGTTSIEK;NLSTKPLFDLK;VPTFWYNLR |
| MGG_06192 | ubiquinol-cytochrome-c reductase activity | 1 | AAQLSLSHQLLPK |
| MGG_06494 | oxidoreductase activity | 1 | VVELFGAPDVLVNSAGIADSNIK |
| MGG_07974 | oxidoreductase activity | 4 | AAGGLTDAIVVAMDPGWVQTR;IVNISSGLGSMAATLSGDGR;QIAAQPDTTVLALTR;TLSNDLSDLEAIK |
| MGG_08164 | protein disulfide oxidoreductase activity | 1 | AAGGGVDATSGTIAALDAIVVK |
|  | **Metabolic** |  |  |
| MGG_00922 | aspartic-type endopeptidase activity | 11 | DLPDITFR;EPGLAFAFGR;FDGILGMGFDR;IQYGSGSMEGFVSNDVMTIGDLK;ITEIPLR;IVPPFYAMVDQK;LIDEPVFAFYLADEK;NLDFAEATK;NLDFAEATKEPGLAFAFGR;SESEVVFGGVNK;YYSIYDLGK |
| MGG_00223 | fructose-bisphosphate aldolase activity | 9 | DWNIETTAK;GVFSELGLKPGVLYGEEVYK;GVSNTNQEASIAGAVAAAHFIR;KPIFFVFHGGSGSGDSEFQEAISYGVIK;LHPELLDK;LLPWLDGMLDADEAFHK;NVYAIPAINVTSSSTIIASLEAAR;SPIILQMSQGGAAYFAGK;VNLDTDLQW |
| MGG_00659 | hydrolase activity, glycosyl bonds | 1 | VLWIDHGIYR |
| MGG_01261 | alpha,alpha-trehalase activity | 8 | AVPLLIK;DNYFPLR;GAIPATNLK;NTVENFLDLVETIGFVPNGAR;SQPPLLSQMVR;VIDIWPSLTR;VPATFGEDDVAWTEIQDLALR;WLSTPSDAVR |
| MGG_01288 | mannose-1-phosphate guanylyltransferase activity | 3 | AIILVGGFGTR;IELRPTSIEQETFPAMVR;IGPNVTIGPNVVVGDGVR |
| MGG_01885 | hydrolase activity, hydrolyzing O-glycosyl compounds | 1 | SPELISLFGYAAK |
| MGG_02653 | 6-phosphofructokinase activity | 3 | AFVIEVMGR;IIATQMGVEAVR;VFVIETQGGK |
| MGG_03245 | isomerase activity | 4 | DQSGPFDWR;ESGPVASLSSDWSGIK;GISAAFVPYGASIANLMIHDK;IVALSLTK |
| MGG_03510 | UDP-glucose:glycoprotein glucosyltransferase activity | 1 | EILLSALR |
| MGG_03687 | dolichyl-diphosphooligosaccharide-protein glycotransferase activity | 1 | DVPLQLLALK |
| MGG_03945 | acetolactate synthase activity; thiamine pyrophosphate | 2 | AASNAVVDQVYALLR;FPVLFLGMR |
| MGG_04860 | hydroxymethylbilane synthase activity | 1 | QVSQVLVER |
| MGG_07197 | ligase activity | 3 | GDVLGLVEDLK;GPTVFSEYYK;NIGAGVVELHK |
| MGG_07331 | transferase activity | 2 | DQLLDDFR;TIQTLLFSATFPDHVK |
| MGG_08624 | C-8 sterol isomerase activity | 1 | AIVGHIVDELR |
| MGG_09922 | beta-N-acetylhexosaminidase activity | 8 | ANAAGEVLWSGR;DLIYTADDIR;GILFDTAR;NLIPWK;QWYPVVNLLR;SEVLQPLLQK;SLGSIFR;SSIGILR |
| MGG_10669 | endonuclease activity | 2 | ILPLTAIR;VATPIPFNPR |
| MGG_11536 | hydrolase activity, hydrolyzing O-glycosyl compounds | 2 | FGTLPLYVRPGTVLLLR;LGPGWFR |
| MGG_11861 | transferase activity | 4 | KWTETTALYSDR;MLDDAGIYVIADLGEPSTSIDR;NFDALVNVMK;TFVDPLADEK |
| MGG_12805 | tryptophan synthase activity | 1 | EHFLSVGGLADGVVVGSQIITTLAK |
| MGG_12939 | hydrolase activity, acting on carbon-nitrogen (but not peptide) bonds; chitin binding | 1 | YTGDLLDIFK |
| MGG_14279 | glutamate-ammonia ligase activity | 3 | DIVEAHYK;HETGAIDTFSYGVANR;VAEEFGAK |
| MGG_15814 | peptidyl-prolyl cistrans isomerase activity | 3 | HVVFGEVLEGYDIVQK;IANSGELPVPEEGIHVEL;IVMGLYGK |
|  | **Transport** |  |  |
| MGG_01557 | lipid transport | 4 | AGESLVIK;SVDLPNEIPPGK;VDLSPNPPK;YTVFADVYTK |
| MGG_02466 | hydrogen ion transmembrane transporter activity | 1 | SGVGIAAMGVLRPDLIVK |
| MGG_02479 | Ran GTPase binding | 2 | AAWSNLVYTDQSVLTFESTEHR;WYALNDVFK |
| MGG_02770 | proton-transporting ATP synthase activity, rotational mechanism | 4 | IEIDNTFEAR;LSILNTSALPAMR;NLLLEGFYALHEPQMQVR;QELLDDIFDAAQK |
| MGG_06209 | phosphate ion transport | 1 | YTNPLLIQSIIPLK |
| MGG_06332 | transmembrane transport | 4 | ATAGATGAVLANALVYPLDIVK;DVIESEDGVFGLWR;SLATIATQPLIVAK;VLFFPGK |
| MGG_07471 | transport | 1 | QGDFVFTVNDIGEYR |
| MGG_09952 | GTPase activity | 6 | HLTGEFEK;IQFDVWDTAGQEK;LVGNPGLEFVAAPALAPPTAEVTAEQAAAYEK;LVLVGDGGTGK;SNYNFEKPFLWLGR;TITFHR |
| MGG_10604 | ubiquinol-cytochrome-c reductase activity | 1 | SAASSIYNLLFR |
| MGG_16213 | microfilament motor activity | 3 | AGMLAFLENLR;LGILSLLDEESR;LVGVTAFNDLLMR |
|  | **Proteolysis** |  |  |
| MGG_03670 | serine-type endopeptidase activity | 6 | AVTVGASALDDSR;AYFSNWGK;GSVANMSLGGGK;SNGSGTMSDVIAGVDFAAK;VGTVGILTDIPK;YLYSAEGGEGVDAYVIDTGTNIDHVDFEGR |
| MGG_04445 | dipeptidyl-peptidase activity | 3 | IPFIADEDLALYQK;QVSPESEHIYDLIISLHK;SFYDGSLLSFK |
| MGG_05989 | aminopeptidase activity; serine-type peptidase activity | 2 | LVVVNVPSGDSSVIR;TIVGALFTEK |
| MGG_07109 | nucleic acid binding | 1 | VGFAFISFADR |
| MGG_08994 | hydrolase activity | 1 | ETPGAVQLLPWLDR |
| MGG_09481 | aminopeptidase activity; leukotriene-A4 hydrolase activity | 4 | AQDSTAYQGVADLLGQVGR;GSVVLELESR;LVFLGSVQDFEQPLSAEQAK;QLGNAYDLIETK |
| MGG_11400 | hydrolase activity, hydrolyzing O-glycosyl compounds | 1 | ITFEDGER |
| MGG_11945 | aspartic-type endopeptidase activity | 3 | AFSLDLR;GEPILGASFLR;IGEAFPGATLDSR |
| MGG_12773 | mannosyl-oligosaccharide 1,2-alpha-mannosidase activity | 1 | IEVFSNIQQLIEER |
| MGG_15423 | serine-type peptidase activity | 1 | FLTTDQALADTAYFAK |
|  | **Development** |  |  |
| MGG_03565 | heat shock protein binding | 1 | DLIQELLQK |
| MGG_05100 | mycelium development | 3 | LDNGLVHASNR;LYVVAR;TTTEFGIGVAK |
| MGG_05449 | structural constituent of ribosome | 2 | MVVPQALR;VFEGVPPPYDK |
| MGG_05663 | serine-type carboxypeptidase activity | 2 | NVYDIR;QDFHIAGESYAGHYIPVFASEILSHK |
| MGG_07753 | structural constituent of ribosome | 4 | AEEILER;FDGIVR;IAVHVTVR;VLEQLSGQTPVYSK |
| MGG_08006 | integral component of membrane | 1 | QFYLATDINR |
|  | **Regulation** |  |  |
| MGG_00884 | enzyme regulator activity | 2 | QPTLEEALHPYFLLVQAVR;YLYYLGR |
| MGG_05865 | chitin binding | 14 | AAPANANPGSFK;EMPELPGDYR;FGLPSATVK;FMSIGGNAPLPWLAPDVGDGFDGIR;FTTAATSTIPR;IKPLDANPK;MVYLEHTGFAVGK;NGVSSGQFVK;TYPNTGGSVMLPLSK;VALYYNGYVTHSVHMGHR;VFFLDK;VFFLDKVESFTQLK;WEMDAMPDGR;YTPPYLSNGK |
| MGG_08741 | nucleic acid binding | 1 | SFFQGFGDITYVK |
|  | **Modification** |  |  |
| MGG_01594 | adenylate kinase activity | 1 | YGFTHLSAGDLLR |
| MGG_03473 | nucleotide binding | 1 | ELVPLFGK |
| MGG_04920 | palmitoyl-(protein) hydrolase activity | 2 | LVPAQYFR;QVGEIVEAANPGTFVYFIR |
| MGG_05063 | phosphoglycerate kinase activity | 17 | AAGFLMK;AGEGDVVLLENLR;AHSSMVGVDLPQK;ELDYFAQALESPK;ELPGVTALSSK;FHIEEEGSAK;IAGAVPTIK;IQLIDNLLDK;IVIIGGGDTATVAAK;LGDIYINDAFGTAHR;LSHVSTGGGASLELLEGK;LSITDVDVK;RPFLAILGGAK;VDFNVPLDDNK;VLDNMAIGNSLFDEAGSK;VVLPVDFITADK;YSLKPVVSELEK |
| MGG_05299 | alkaline phosphatase activity | 5 | AVLDFLER;EVIEYDNAFK;NLIFMVSDGMGPASLSLTR;SQNDEVAAQEVGEGPLGR;VVDLMFGGGR |
|  | Response stress |  |  |
| MGG_06459 | nucleoside-triphosphatase activity | 1 | LAVLILR |
|  | **Unknown** **function** |  |  |
| MGG_15670 |  | 1 | IALGPLEYVAATAR |
| MGG_17961 |  | 1 | ITEILK |
